# Sequence Analysis of the Human Virome in Febrile and Afebrile Children

**DOI:** 10.1371/journal.pone.0027735

**Published:** 2012-06-13

**Authors:** Kristine M. Wylie, Kathie A. Mihindukulasuriya, Erica Sodergren, George M. Weinstock, Gregory A. Storch

**Affiliations:** 1 The Genome Insititute, Washington University School of Medicine, Saint Louis, Missouri, United States of America; 2 The Department of Pediatrics, Washington University School of Medicine, Saint Louis, Missouri, United States of America; Institut Pasteur of Shanghai, Chinese Academy of Sciences, China

## Abstract

Unexplained fever (UF) is a common problem in children under 3 years old. Although virus infection is suspected to be the cause of most of these fevers, a comprehensive analysis of viruses in samples from children with fever and healthy controls is important for establishing a relationship between viruses and UF. We used unbiased, deep sequencing to analyze 176 nasopharyngeal swabs (NP) and plasma samples from children with UF and afebrile controls, generating an average of 4.6 million sequences per sample. An analysis pipeline was developed to detect viral sequences, which resulted in the identification of sequences from 25 viral genera. These genera included expected pathogens, such as adenoviruses, enteroviruses, and roseoloviruses, plus viruses with unknown pathogenicity. Viruses that were unexpected in NP and plasma samples, such as the astrovirus MLB-2, were also detected. Sequencing allowed identification of virus subtype for some viruses, including roseoloviruses. Highly sensitive PCR assays detected low levels of viruses that were not detected in approximately 5 million sequences, but greater sequencing depth improved sensitivity. On average NP and plasma samples from febrile children contained 1.5- to 5-fold more viral sequences, respectively, than samples from afebrile children. Samples from febrile children contained a broader range of viral genera and contained multiple viral genera more frequently than samples from children without fever. Differences between febrile and afebrile groups were most striking in the plasma samples, where detection of viral sequence may be associated with a disseminated infection. These data indicate that virus infection is associated with UF. Further studies are important in order to establish the range of viral pathogens associated with fever and to understand of the role of viral infection in fever. Ultimately these studies may improve the medical treatment of children with UF by helping avoid antibiotic therapy for children with viral infections.

## Introduction

Viruses are thought to be the primary cause of unexplained fever in children under 3 years old, a common problem that results in medical visits and in some cases hospitalization [1], [2]. With the implementation of several vaccines against bacterial infections, the frequency of bacterial infections is low [3]–[6]. While viruses are suspected to be the cause of fevers in children with no documented bacterial infection, in clinical practice, tests for viruses are often not performed, and consequently no cause for the fever is determined. In the absence of a clear diagnosis, antibiotics are often prescribed (Colvin et al., manuscript submitted). A comprehensive analysis of viruses in children with fever could improve our understanding of the causes of unexplained fevers (UF) and ultimately lead to modifications of the treatment of children with UF, including restricted use of antibiotics.

Next generation sequencing technologies have been used successfully for viral metagenomic analyses [7]–[11] and discovery of novel viruses [13]–[15]. The Roche 454 platform has been favored over the Illumina GAIIX platform because its longer read lengths are argued to be advantageous for detecting more remote sequence homologies with known viruses. However, the sequence depth per unit cost is much greater using the Illumina platform, and greater sequencing depth would presumably favor the detection of rare virus sequences in metagenomic samples. In this study we sought to develop a sensitive, cost-effective method for characterizing the human virome, the viral component of the human microbiome, to be applied to the analysis of the virome in samples from febrile and afebrile children.

We analyzed 176 plasma and nasophyaryngeal swab (NP) samples from children with UF (febrile) and afebrile children by high-throughput sequencing using the Illumina platform. Using sequencing for the analysis of viruses associated with UF has several advantages. First, unlike targeted PCR assays, sequencing can detect unexpected and novel viruses. Second, sequencing can provide additional information such as virus subtype or sequence variation from reference genomes, adding detail to the understanding of the viruses present. Using the protocol we developed, we detected viruses in 86% of plasma samples and 63% of NP samples from febrile and afebrile children. Furthermore, distinctions between the viromes of febrile and afebrile groups were observed. The assessment of known viruses and initial identification of potentially novel viruses using short-read Illumina sequencing moves us toward a more complete understanding of the human virome and its role in health and disease.

## Results

### Detection of Known Viruses

The goal of our study was to use high-throughput, deep sequencing analysis methods to characterize the human virome in large sample sets. Previous studies demonstrated improved virus detection using deeper sequencing on the 454 pyrosequencing platform compared to Sanger sequencing [16] and we sought to determine whether the greater sampling depth possible with the Illumina GAIIX platform would provide a further improvement. This required development of methods for processing the small clinical sample amounts for Illumina sequencing, computational pipelines for management of the large number of Illumina reads from multiple samples, and accurate detection of viruses from the short Illumina reads.

In preliminary experiments, clinical samples with known viruses were used to develop these methods. Plasma samples were identified that were PCR positive for either an enterovirus (a group of relatively small, single-stranded RNA viruses) or human herpesvirus 6 (HHV-6, a large, double-stranded DNA virus). The total nucleic acid (see Methods) from each sample was amplified in two independent experiments, and the replicates were sequenced on the 454 and Illumina platforms. In the initial Illumina experiment, 75-base, paired-end reads were generated (Table 1). The enterovirus sample, in which virus was detected by real-time PCR with an average Ct value of 30.6 from multiple experiments, showed viral reads with both 454 and Illumina platforms (Table 1). A single HHV-6 read was found in one of the 454 replicates, but reads were found in both Illumina replicates (see Methods for sequencing analysis pipeline). In both the enterovirus and HHV-6 samples, the number of virus reads was higher on the Illumina platform compared with the 454 platform, indicating that increased depth of sequencing strengthened the virus signal. These results encourgaged us to improve the Illumina library construction and sequencing protocol. Specifically, we increased the length of fragments from 300–400 base pairs (gel purified) to 300–800 base pairs by minimizing shearing. Second, the Illumina read-length was increased to 100 bases to facilitate identification of viral sequences, especially those that were divergent from reference genomes. 3- to 13-fold more HHV-6 and enterovirus sequences were detected with this modified protocol (Table 1). Based on this result, we reduced the read depth for subsequent analyses to 3–5 million per sample, which facilitated processing large numbers of clinical samples. The improved protocol sampled more broadly across the enterovirus genome with 65% of the reads assembling into small contigs covering 1907 bases of the enterovirus genome. In contrast, more than 97 percent of the 1473 reads from the first experiment were concentrated in two contigs that covered only 550 bases. We next sought to apply the 100-base read-length protocol to large-scale analysis of samples from afebrile children and those with UF.

**Table 1:** Detection of viruses with next generation sequencing.

			454 reads		Illumina (75 base) reads			Illumina (100base) reads		
Sample	PCR	Replicate	Total	Viral	Total	Viral	% genome length covered	Total	Viral	% genome length covered
9008	Enterovirus	A	58,924	11	34,612,722	1473	7.50%	4,792,380	342	42.3%
9008	Enterovirus	B	45,625	2	35,760,754	419	–	–	–	–
9022	HHV-6	A	62,361	1	36,629,654	4	–	3,931,804	8	–
9022	HHV-6	B	50,866	0	34,933,530	7	–	–	–	–

### Sequencing and Analysis of Samples from Febrile and Afebrile Children

Nasopharyngeal (NP) swabs and plasma samples from children 2–36 months of age with fever without an apparent source and afebrile controls from the same age group (Table 2) were sequenced on the Illumina GAIIX. The samples that were sequenced were a subset of those included in a PCR-based analysis that tested for 15 genera of known viral pathogens, 12 in NP samples and 7 in plasma samples (Table 3) (Colvin et al., manuscript submitted). The median number of sequences produced per sample was approximately 4.4 million (Figure S1). The median and mean numbers of reads for the NP febrile, NP afebrile, and plasma febrile groups were each greater than 4 million. However, the number of reads from the plasma afebrile group was lower, with a median of 2.4 million and mean of 3.2 million reads, which may indicate less nucleic available for amplification and sequencing in plasma from healthy children.

**Table 2:** Samples.

	Afebrile	Unexplained fever (UF)
**Nasopharyngeal swab**	81	50
**Plasma**	22	23

**Table 3:** Virus genera screened by PCR.

Genus	PCR assay targets	Samples assayed by PCR
Alphacoronavirus	229E, NL63	NP
Betacoronavirus	OC43, HKU1	NP
Bocavirus	Human bocaviruses	NP and Plasma
Enterovirus	Enteroviruses, Rhinoviruses	NP – Enteroviruses and RhinovirusesPlasma - Enteroviruses
Erythrovirus	Parvovirus B19	Plasma
Influenza A Virus	Influenza A, H1, H3,	NP
Influenza B Virus	Influenza B	NP
Mastadenovirus	Human adenoviruses	NP and Plasma
Metapneumovirus	Human metapneumovirus	NP
Parechovirus	Human parechoviruses	Plasma
Pneumovirus	RSV A, RSV B	NP
Polyomavirus	JC, BK, WU, KI	NP – WU and KIPlasma – All 4
Respirovirus	Human parainfluenza virus (HPIV)-1, HPIV-3	NP
Roseolovirus	Human Herpesvirus (HHV)-6, HHV-7	Plasma
Rubulovirus	HPIV-2, HPIV-4	NP

**Figure 1:**
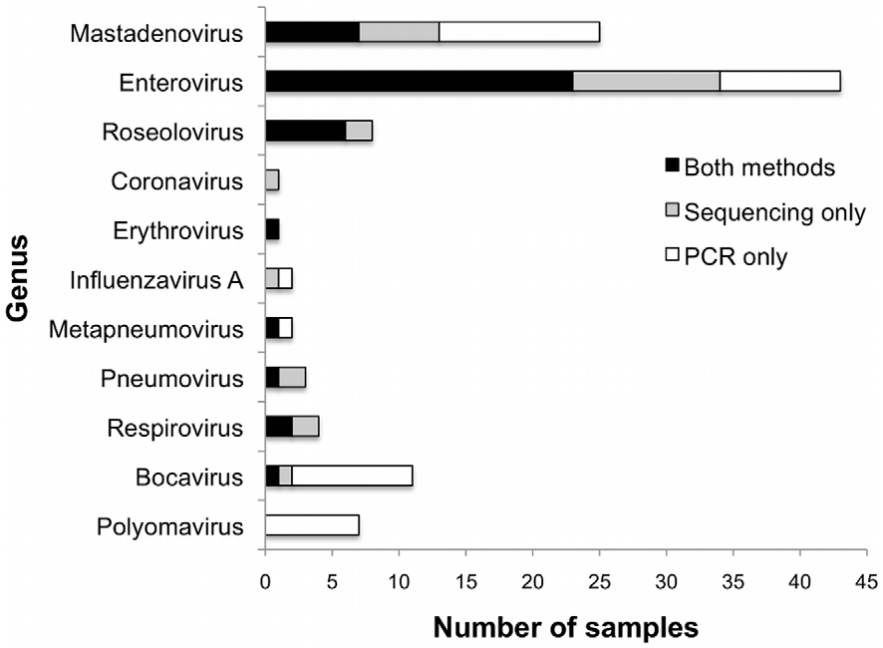
Comparison of sequencing and PCR results. The number of samples in which each virus was detected by PCR (white bars), sequencing (gray bars), or both (black bars) is shown.

**Figure 2:**
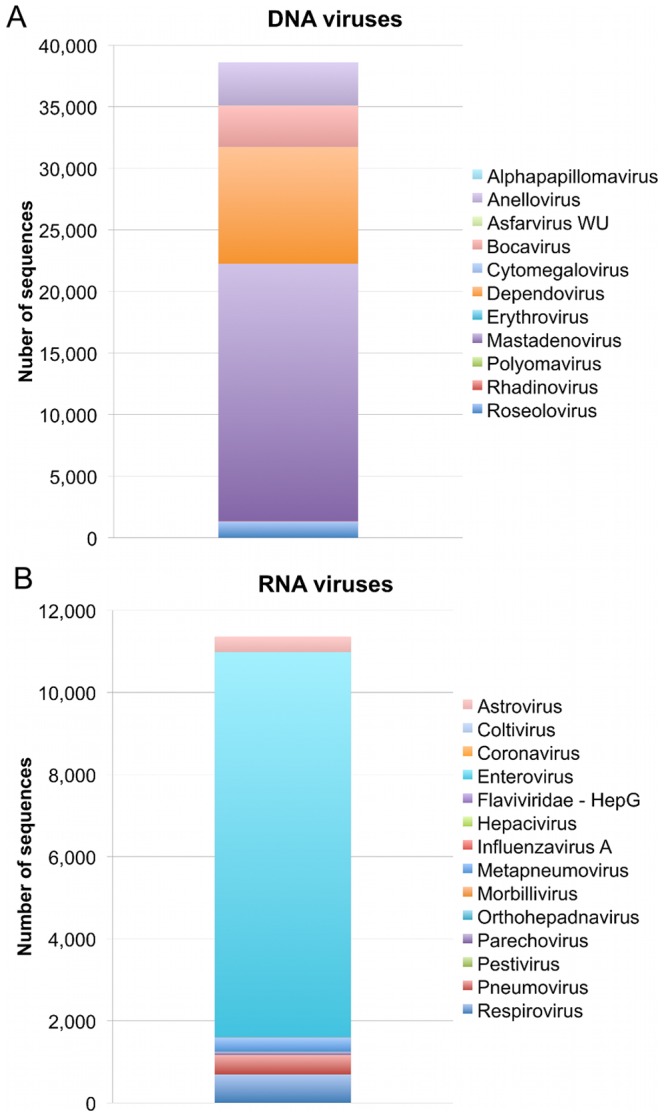
Sequence analysis identifies a variety of viruses in samples from febrile and afebrile children. Analysis of 176 plasma and NP samples on the Illumina GAIIX and HiSeq 2000 platforms identified approximately 50,000 sequences with similarity to 25 known (A) DNA and (B) RNA virus genera.

Sequences from 11 of the 15 viral genera in the PCR panel were detected in samples that had been tested by both sequencing and PCR, allowing for the comparison of methods (Figure 1). Only Betacoronavirus, Influenza B virus, and Rubulovirus were not found by either method. Parechovirus was found in NP samples by sequencing, but NP samples were not screened by PCR for this virus. Likewise, blood samples were not tested by PCR for respiratory viruses. These 11 viral genera were detected in 90 samples. In 39 instances the same virus was detected by both methods, 25 were found only by sequencing, and 42 were found only by PCR (Figure 1). Beyond the 11 genera targeted by PCR, sequences from a variety of both DNA and RNA viruses were detected by sequencing (Figure 2 A and B, respectively). Nearly 50,000 sequences with similarity to 25 known viral genera were identified.

### Comparison of Sequencing and PCR Results for the Most Commonly Detected Viruses

**Figure 3:**
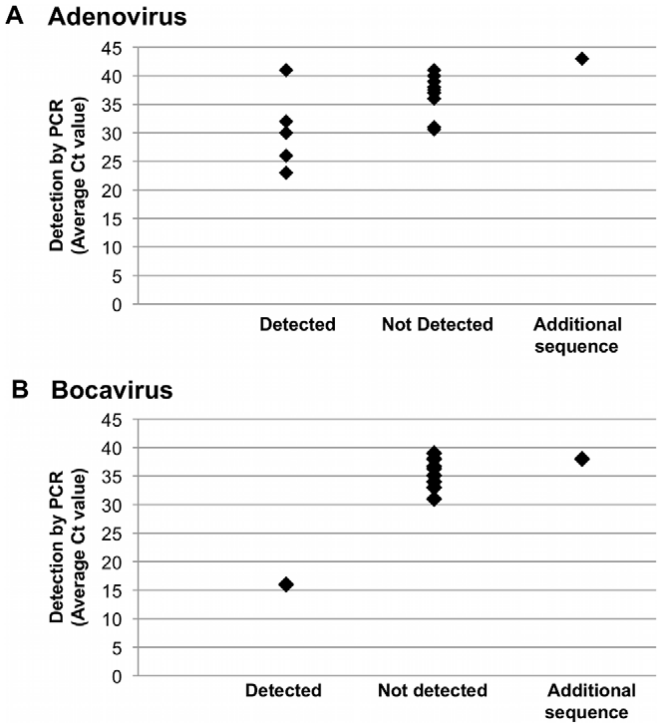
Comparison of sequencing results with Ct values from real-time PCR assays. The average Ct values from real-time PCR assays for (A) adenovirus and (B) bocavirus are graphed. Each PCR experiment was repeated at least twice per sample. Samples are grouped from left to right according to whether they were detected by sequencing, not detected by sequencing, or only detected when the number of sequence reads was increased to 21 or 51 million sequences for A and B, respectively.

Mastadenoviruses, enteroviruses, and roseoloviruses were frequently detected by both sequencing and PCR, which allows a thorough comparison of the methods. Mastadenoviruses (referred to as adenoviruses) were detected in 19 samples by PCR, and 5 of those were confirmed by sequencing (Figure 1). For those samples in which an adenovirus was detected by sequencing, the Ct values in the real-time PCR assay tended to be lower (average Ct = 29.1) compared with those samples in which an adenovirus was not detected (average Ct = 37.8) (P = 0.0023), suggesting that deeper sequencing would be required to detect the low levels of virus present in these samples (Figure 3A). When we sequenced one NP sample (sample 9021-581), which had a Ct of 42.6 and was originally negative for adenovirus by sequencing, to a read depth of greater than 21 million reads, we were able to detect 2 adenovirus reads, consistent with the PCR result. However, for the plasma sample from the same subject (sample 9021-895), which had a Ct of 37.5, adenovirus was still not detected after generating more than 50 million reads. Interestingly, 192 and 9,003 adeno-associated virus sequences were detected in these NP and plasma samples respectively, indirectly supporting the presence of adenovirus detected by PCR. The detection of the adeno-associated virus demonstrates that the unbiased nature of shotgun sequencing can reveal the presence of additional viruses not evaluated by PCR.

Enteroviruses (enterovirus and rhinovirus species) were detected in 32 samples by PCR, of which 21 were also detected by sequencing (Figure 1). Different commercial PCR assays were used for plasma and NP samples. For the plasma samples, a real-time PCR assay from Cepheid run on the SmartCycler thermal cycler (Cepheid, Sunnyvale, CA) was used (Colvin et al., manuscript submitted). Enterovirus sequences were detected in each of the 5 plasma samples that were positive for enterovirus by PCR. The NP samples were assessed using the xTAG Respiratory Virus Panel, produced by Luminex or the Multicode PLx Respiratory Virus Panel produced by Eragen Inc (Madison, WI) (Colvin et al., manuscript submitted). Of the 27 PCR-positive NP samples, rhinovirus or enterovirus sequences were detected in 16. Although the MFI read out of the Luminex assay is not strictly quantitative, the average MFI for samples with rhinovirus or enterovirus detected by sequencing was significantly higher than the average MFI of those samples missed by sequencing (P = 0.0193), suggesting that enteroviruses are present at low levels in some samples and would require deeper sequencing for detection. In support of this, sequencing one sample to a read depth of greater than 20 million reads produced 2 previously undetected rhinovirus reads (sample 9031-591, MFI 1500).

For the roseoloviruses, HHV-6 and HHV-7, sequences were detected in every sample that was positive by PCR and also in 2 additional samples that were negative by PCR (Figure 1). The sensitivity of sequencing appeared to be high, possibly because the large genome size of the roseoloviruses allows the virus to be sampled efficiently during sequencing. The roseolovirus assay is a conventional (not real-time) PCR assay that is not quantitative, so the level of virus present in these samples is unknown. Futhermore, roseoloviruses were found in 5 NP samples by sequencing, while NP samples were not screened by PCR for these viruses.

As mentioned above, there were 25 examples of adenovirus, enterovirus, roseolovirus and a number of less frequently detected viruses that were found by sequencing but not by PCR (Figure 1). In some cases, significant sequence similarity to the reference genome was found at the amino acid but not nucleotide level, suggesting that these might be novel or divergent virus sequences not detected by the PCR assay. Future analyses will be aimed at characterizing viruses with remote sequence similarities to reference genomes and validating viruses detected by sequencing but not by PCR.

Polyomaviruses (WU and KI) and bocaviruses were detected by PCR, but rarely or not at all by sequencing (Figure 1). We did not detect any polyomavirus sequences in any of the samples that were positive by PCR. This includes one sample in which KI was detected with a Ct of 39.2 that was sequenced to a depth of greater than 21 million reads. WU and KI polyomavirus Ct values were relatively high, with all but one above 30. The sensitivity of the WU PCR assay is 7 plasmid copies per reaction and that of the KI assay is 50 plasmid copies [17]. Thus, the level of the viruses present in the samples may be relatively low, and this fact, coupled with the small, 8 kb genome size suggests that little nucleic acid is available for detection. Bocavirus was detected 10 samples by a sensitive PCR assay that can detect as few as 5 plasmid copies of the target [18] (Figure 1). During initial sequencing of a sample from a febrile child with respiratory symptoms, not included in the UF group, the 5 kb bocavirus genome was detected by sequencing. This sample had higher levels of bocavirus by PCR than other samples (sample 9105-663, Ct 16.17). A second sample containing less bocavirus (sample 9013-573, Ct 37.59) was sequenced to a read depth of greater than 50 million reads, yielding 4 bocavirus reads (Figure 3B). These results show sequencing enables detection of viral genomes present at low levels, but requires additional optimization to target rare smaller genomes.

**Figure 4:**
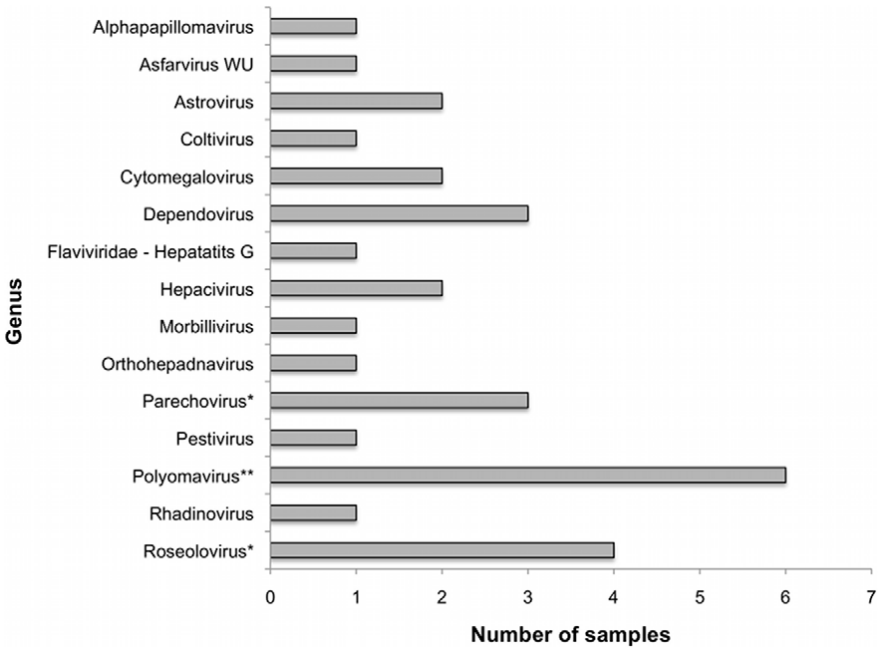
Viruses detected by sequencing that were not screened by PCR. The bars represent the number of samples in which each virus was detected by sequencing. *Indicates viruses that were not assayed by PCR in NP samples. **Indicates a virus that was not assayed by PCR but belongs to a genus with members that were.

Many samples contained sequence reads that aligned to reference genomes from viruses that were not included in the PCR panels (Figure 4). NP samples positive for parechoviruses and roseoloviruses are included in this figure because those viruses were only assessed by PCR in plasma samples. The polyomavirus-positive samples contained SV40-like sequences, which were not included in the polyomavirus PCR assays. Each of the viral sequences represented in Figure 4 were detected in samples from febrile children, with the exception of the alphapapillomavirus and some of the roseolovirus sequences. Some of the viruses detected are known to be pathogenic (parechovirus), while the pathogenic significance of others remains undetermined (hepatitis G virus). Further validation and characterization of some of these unexpected viruses may reveal the presence of novel viruses with remote homologies to the known references or a previously unknown role for a virus in febrile illness.

In plasma and NP samples from one febrile subject with UF, we detected astrovirus sequences. The sequences from the plasma sample assembled into contigs that spanned 55% of the recently discovered astrovirus MLB2 genome. Astroviruses have previously been detected in stool samples and are associated with diarrhea. However, this is the first time an astrovirus has been detected in either NP or plasma samples. No other pathogen was detected in these samples, suggesting the astrovirus may have been the cause of the subject’s fever. We have subsequently extended the sequence of the capsid gene of this MLB2 virus, confirming its presence by both sequencing and PCR of RNA from the plasma sample and allowing us to compare this virus to another MLB2 isolate [19].

One concern about using 100-base reads for virome analysis is that shorter read lengths may not permit the discovery of novel viruses. Therefore, we asked whether we would have detected the astrovirus sequences had MLB2 or closely related MLB1 not been previously discovered. In fact, 162 reads from this sample have amino acid sequence similarity to other human, avian, and mammalian astroviruses (Table 4) and, therefore, this method would have been successful at detecting a novel virus.

**Table 4:** Illumina sequences with remote homologies to astroviruses.

Virus	Number of sequences
Astrovirus from dog feces	14
Bat astrovirus	47
Bottlenose dolphin astrovirus	1
California sea lion astrovirus	4
Human-mink astrovirus	3
Human astrovirus	59
Rat astrovirus	17
Sheep astrovirus	2
Swine astrovirus	14
Turkey astrovirus	1

The top alignment from tblastx, excluding MLB1 and MLB2, is reported if the alignment was to an astrovirus.

In contrast to the samples in which viruses were present at low levels with only a few sequence reads detected, many samples yielded sufficient virus sequences to provide more detailed information about the virus in the sample. In some cases the genome coverage was sufficiently deep and broad for contigs to be assembled that covered significant portions of the genome (Table 5). The genome coverage allowed us to show that the human bocavirus was type 1, with 99% identity to known reference genomes. Likewise, we were able to determine that one of the rhinoviruses we detected was most similar to the recently discovered group C rhinovirus QPM [20].

**Table 5:** Genome coverage.

Genome	Contigs	Input sequences	Sequences incorporated into contigs	Smallest contig	Largest contig	Genome size	Coverage
**Human bocavirus**	5	2733	2344	100 nt	1886 nt	5299 nt	92.6%
**Respiratory syncitial virus**	24	2588	2070	102 nt	1882 nt	15,191 nt	58.4%
**Human rhinovirus QPM**	2	7159	6744	798 nt	5962 nt	6948 nt*	94.5%*
**Human parainfluenza virus**	10	189	107	105 nt	803 nt	15,462 nt	14.2%

* Full genome sequence not available. Largest Genbank sequence used.

### Do Viral Sequences Correlate with Fever Without a Source?

**Figure 5:**
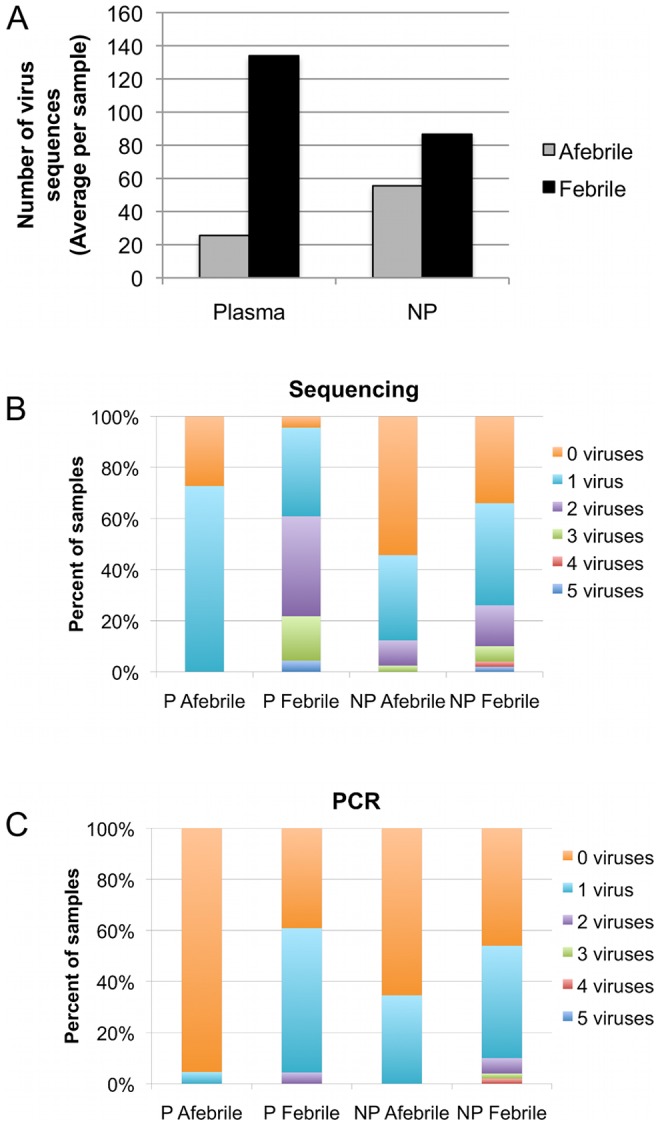
Febrile children have more viral sequences from a greater range of viruses than do afebrile children. The number of sequences was scaled to 3 million per sample before comparisons were made between groups. (A) The average numbers of viral sequences found in plasma and NP samples from the subjects are represented by gray bars for samples from afebrile children and black bars for samples from febrile children. The percentage of samples in each group for which 0, 1, 2, 3, 4, or 5 viruses was detected is plotted for (B) sequencing data and (C) PCR data.

To compare afebrile children and children with UF, the number of sequence reads was normalized to 3 million per sample. After the adjustment, samples from children with UF had 1.5- to 5-fold more viral sequences than samples from afebrile children in NP and plasma samples, respectively (Figure 5A). Although sequencing is not strictly quantitative, the number of sequences generated was inversely correlated with Ct values from the real-time PCR assays (Figure S2), which suggests the number of virus reads correlates with the amount of viral genomic material is present. More than one viral genus was found in some samples, and samples from children with fever had a greater number of viruses compared to those from afebrile children (Figure 5B). No plasma sample from an afebrile child had more than 1 viral genus detected, compared to 2 to 5 genera detected in 61% of the plasma samples from febrile children (Figure 5B). The difference in the percentages of samples with multiple viruses was not as striking in NP samples from febrile and afebrile children, although only 12% of samples from afebrile children had 2 or more viruses compared with 26% of samples from febrile children (Figure 5B). In all groups, sequencing detected multiple viral genera in a larger proportion of the samples than did directed PCR assays (Figure 5C).

**Figure 6:**
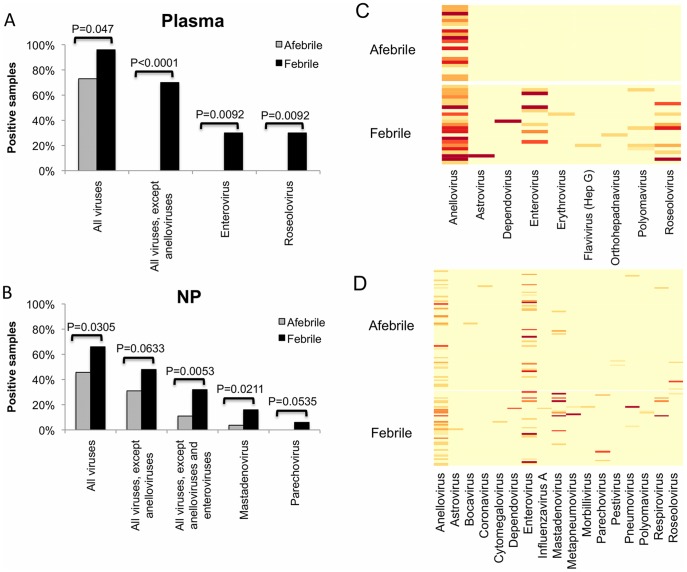
Prevalence of viruses in samples from febrile compared with afebrile children. The total number of reads per sample was scaled to 3 million to make samples more comparable, and all counts of ≥1 virus sequence were reported. The percent virus-positive samples are graphed for plasma and NP samples, respectively (A and B). P-values were determined using Fisher’s exact test. Heatmaps representing the number of virus reads for each virus detected (x-axis) and each sample evaluated (y-axis) are presented for plasma and NP samples, respectively (C and D). The light yellow area is 0 reads, with more intense red representing larger numbers of reads.

More plasma samples from febrile children were positive for viral sequences than were samples from afebrile children. Anellovirus sequences were the only group found in the plasma from afebrile children. They were found in 80% of all plasma samples, and there was no significant difference between the presence of anellovirus sequences in febrile and afebrile children (P = 0.2837, Fisher’s Exact Test). The presence of anelloviruses is not surprising, as they infect the majority of children by 1 year of age and establish chronic infections that are detectable in the blood of healthy individuals [15], [21]–[22]. By removing the ubiquitous anellovirus sequences from the analysis the difference between the febrile and afebrile groups became even more striking (Figure 6A). Most viruses were detected in only a few samples, so differences between the febrile and afebrile groups were not statistically significant for individual viruses in this limited sample set. However, the enterovirus and roseolovirus sequences were more likely to be found in the febrile subjects than the afebrile subjects (Figure 6A), consistent with their roles as pathogens that can cause fever.

Viral sequences were also detected more commonly in NP samples from febrile children compared with those from afebrile children (Figure 6B). Again, anellovirus sequences were ubiquitous. Enterovirus sequences were found in similar proportions in samples from febrile and afebrile children. Excluding the ubiquitous anelloviruses and enteroviruses, the less common viral sequences were detected more frequently in the febrile subjects compared to the afebrile subjects (Figure 6B). Specifically, adenovirus and parechovirus were more commonly associated with NP samples from febrile children (Figure 6B). These data indicate that viruses are more commonly associated with samples from febrile children and suggest that viruses are the cause of many fevers in young children for which a source is not determined.

Sequences from febrile children revealed a greater range of viral genera compared to sequences from afebrile children. The difference was most striking in plasma, with sequences from 9 genera found as a result of screening the 23 samples from febrile children and 1 genus found as a result of screening the 22 samples from afebrile children (Figure 6C). In NP samples, sequences from 14 genera were detected by screening the 50 samples from subjects with UF compared with 10 genera detected by screening the 81 samples from afebrile subjects (Figure 6D). These data indicate that fever is likely associated with a broad range of viruses, and further studies with larger sample sizes may be important for elucidating the roles of particular viruses in febrile illness.

## Discussion

Although it has long been suspected that virus infection is the cause of many unexplained fevers in children under 3 years old, this is the first comprehensive analysis of viruses in samples from children with UF and controls using deep sequencing. We show that more viral sequences from a greater diversity of viruses are found in plasma and NP samples from children with UF than in corresponding samples from afebrile children, which supports the idea that viruses are the cause of many of these unexplained fevers. Children with UF are frequently hospitalized or treated with antibiotics without a positive test for a bacterial infection. The evidence we provide indicates that viruses are commonly associated with UF, and further studies should be done to confirm and elaborate on their role in this clinical syndrome. Ultimately, it would be helpful to identify specific clinical features or tests that could aid diagnosis of virus infection to improve the treatment of children with UF and minimize the unnecessary use of antibiotics.

As expected, the virome of the nasopharynx, which is directly exposed to the environment, is much more complex than the virome detected in plasma. Some viruses found in NP swabs were detected in both febrile and afebrile children. Of particular interest are the *Enterovirus* sequences, which include rhinoviruses that are known to cause colds. The presence of an enterovirus or rhinovirus in an NP sample from a child with fever would likely lead a physician to conclude that the enterovirus or rhinovirus was the cause of the fever, but we show that *Enteroviruses* are equally prevalent in the NP samples of afebrile children. These data suggest that in a microbial habitat that is exposed to the environment, the presence of a known pathogen should be interpreted with caution. These data also suggest that we are exposed to a number of known pathogens without showing symptoms of infection, either because the presence of the virus is transient or the particular virus species or strain does not cause symptoms. These observations indicate the importance of future experiments to evaluate the microbiome of the airways over time to look for indicators that a viral infection will become symptomatic, such as correlation of symptoms with specific viral subtypes, correlation with specific biomarkers, or shifts in the larger microbial community structure.

The detection of viruses in the plasma has different implications than in NP samples. Plasma is not generally exposed to the environment, so the presence of a known viral pathogen in the plasma is most likely the result of a disseminated infection. While this study was not designed to determine causation of fever, the complete absence of known viral pathogens in the plasma of afebrile subjects suggests the viral pathogens detected in the plasma of febrile subjects were the sources of their fevers. While it is more invasive to collect blood than other samples, these data suggest blood samples may provide clearer assessment of viruses that are directly associated with disease in contrast to NP samples where viral pathogens are detected in asymptomatic individuals. Additional studies will need to be done to confirm these ideas. Other viruses, such as anelloviruses, are present chronically in the plasma of healthy people. It remains to be determined what kind of effects long-term exposure to these viruses has on the immune response and human health.

This study could be expanded in several ways in order to better characterize the role of viruses in UF, including detecting viruses in children in whom no viruses have been detected thus far. The first would be to include additional sample types, such as stool. The second would be deeper sequencing of samples, particularly plasma, in which the presence of virus sequences are most likely to be clinically significant. We confirmed that additional sequencing improved virus detection of low abundance virus sequences, and as sequencing costs decrease and analysis tools improve it may be practical to generate and analyze 10 times the number of sequences for each sample to enhance virus detection. It is notable that the use of the Illumina platform in this study enabled the detection of many rare virus sequences, which would likely have been missed using sequencing platforms that generate fewer sequencing reads per unit cost. The third way to improve the study would be further examination of existing sequence data for novel viruses, focusing especially on samples from febrile children with no pathogen detected.

Virus discovery using high-throughput sequencing methods has been very productive in recent years [12]–[14], [23]–[25]. While short-read Illumina sequencing has not been widely adapted for virus discovery in metagenomic samples to date, our findings suggest that this 100-base platform can be applied to virus discovery. For example, the sequences we obtained from the recently discovered astrovirus MLB2 and rhinovirus QPM would have allowed discovery of those viruses based on alignment to other more remotely related reference genomes. In addition, the depth of sequencing gained using the Illumina platform gives the advantage of detecting more virus sequences compared to the 454 platform, which could be advantageous by allowing alignment over different parts of a reference genome, some of which may be more conserved, and by generating enough sequences to enable longer, contiguous sequences to be assembled for further analysis.

An important outcome of this study is to show that deep, Illumina-based sequencing has at least two advantages over targeted, PCR-based assays for the assessment of viruses in clinical samples. First, sequencing does not require prior knowledge of which viruses might be in the sample, thus allowing the detection of unexpected and novel viruses. Second, sequencing can often provide information such as virus subtype or sequence variation from reference genomes, which adds detail to our understanding of the viruses present. Our study illustrates both of these advantages. First, we identified viruses that would not have been routinely queried by PCR assays for known pathogens. For example, we detected the astrovirus MLB2 in plasma and NP samples from a febrile child, which were subsequently confirmed by PCR in both samples ([19] and data not shown). Because no other cause of the fever has been detected, these data suggest MLB2 is the cause of this subject’s fever and further examination of the role of this virus in pediatric fever is warranted.

The second advantage of sequencing, the ability to determine virus subtype or sequence variation from reference genomes, is also evident in our study. For example, we were able to identify specific types or subtypes or strains of rhinovirus and bocavirus. Notably, this can often be accomplished without sequencing most of the viral genome. In the case of HHV-6, all of the positive plasma samples were determined to be serotype 6B, even though 4 of the 8 samples had fewer than 15 HHV-6 sequences. We were also able to make distinctions between anellovirus species TTV, TTMDV, and TTMV with as little as one read. In future studies we will examine how different virus species and subtypes correlate with clinical symptoms.

One challenge in analyzing the virome in metagenomic samples is the speed of alignment tools available. Aligners designed for large data sets with short sequences generally gain processing speed by sacrificing the ability to identify sequences that differ more than slightly from the reference genome. Thus, many of these very fast aligners cannot be used effectively for analysis of virus sequences, which frequently differ considerably from their most closely related reference sequences. We are implementing new tools to be used for virome analysis that improve the speed of nucleotide and amino acid sequence alignments while retaining most of the sensitivity, which will allow the efficient analysis of a greater number of sequences. A second challenge for virome analysis is the use of a more inclusive reference database (such as NCBI’s NT) because this would allow identification of more virus sequences based on sequence similarity; however, alignment results from a large database can be problematic for several reasons: (a) taxonomy can be irregular causing computational problems and (b) some of the viral entries contain sequences from the human genome or bacterial cloning vectors, which cause false positive alignments. We have addressed these problems in the present study by manually reviewing the data, but our goal is to develop an easily updated, semi-curated database that would minimize these problems. Future versions of this analysis protocol will be improved with faster alignment tools and improved databases.

This study of deep sequencing of samples from febrile and afebrile children indicates that viruses are frequently detected in both groups, but with greater frequency and diversity in the samples from children with fever of unknown cause. A causal role for these viruses would have important implications for the medical treatment of these children, since the children would not require antibiotic therapy. In evaluating viral causes of fever, sequencing appears to be advantageous in that it frequently reveals the presence of multiple viruses in a given sample, including unexpected viruses. Highly sensitive and specific PCR assays for a subset of viruses complement the sequencing analysis. As sequencing continues to become less expensive and the speed of computational tools improves, it is possible that its sensitivity could match that of PCR. This could lead to a powerful diagnostic approach: rapid, unbiased sequence analysis of the microbiome in patient samples, which could identify potentially pathogenic viruses and other microbes, followed by confirmation of the results using highly targeted and extremely specific PCR assays.

## Methods

### Ethics Statement

Samples were collected from human subjects using a protocol that was approved by the Washington University Human Research Protection Office. Written informed consent was obtained from the parents or legal guardians of all subjects.

### Sample Collection

The subjects included were febrile and afebrile children 2 to 36 months of age seen at St. Louis Children’s Hospital. The group of febrile children was comprised of patients seen in the emergency room who had fever without an obvious source. In order to be included in the study, the physicians must have elected to obtain blood for testing. The afebrile group was comprised of children undergoing surgery. NP swabs and plasma samples were collected as described (Colvin et al., manuscript submitted). NP swabs were collected by inserting flocked swabs into the nasopharyngeal area, rotating the swab, and holding the swab in place for 10–15 seconds to increase specimen collection. Swabs were submerged in Universal Transport Medium (Copan), and the shafts of the swabs were cut or broken off and discarded. The medium containing the swab was briefly vortexed, and then the swabs were removed without wringing out any absorbed medium. Tubes were centrifuged, and the supernatant was aliquotted and frozen at −70°C. Total nucleic acid was extracted using the Qiagen BioRobot M48 the Roche MagNA Pure automated extractor for NP and plasma samples, respectively.

### Sample Preparation and Sequencing

For samples from afebrile and febrile children, sequencing libraries were prepared to look for DNA and RNA viruses. DNA and RNA were prepared as previously described [26]–[27]. In brief, using total nucleic acid templates, RNA was primed with Primer A for reverse transcription. Sequenase DNA polymerase was used for second strand synthesis. DNA and RNA fragments were amplified with Primer B for 40 cycles. Samples were sequenced on the Roche 454 GS FLX Titanium or Illumina GAIIX. For samples in which additional sequencing reads were generated, the Illumina GAIIX or Illumina HiSeq 2000 was used (Figure S3). The SRR accession numbers for the sequence data are provided in Figure S4.

### Sequence Analysis

A pipeline was developed for the analysis of large numbers of short sequence reads. This was adapted from that used for the analysis of 454 sequences, which used BLASTn and tBLASTx [28] to align sequences to references in the NT database (ftp://ftp.ncbi.nlm.nih.gov/blast/db/), followed by a manual review of the viral alignments. The details of the protocol for analysis of short reads follow. After removal of primer sequences, completely identical sequences were collapsed into a single representative sequence to minimize the number of sequences to be analyzed. Low complexity sequences were then masked using Dust [29]. Sequences with greater than 20 N nucleotides (either from sequencing error or as a result of Dust) were removed. Human sequences were identified for removal by aligning sequences to the Genome Reference Consortium’s human build 36 (http://www.ncbi.nlm.nih.gov/projects/genome/assembly/grc/data.shtml) including unplaced, human mitochondrial, and 5.8 s, 18 s, and 28 s rDNA sequences using cross_match [30] with the following alignment parameters: minscore 70, bandwidth 3, penalty -1, gap_init -1, gap_ext -1, masklevel 0. Non-human sequences were aligned to a metagenomic database consisting of all virus and phage sequences in NCBI NT plus full genomes from other microbes including bacteria, archaea, and small eukaryotes (Mitreva, et al., unpublished). Cross_match was used with the same parameters used for the human alignments. Any sequences that were unaligned using nucleotide alignment were then aligned to NR (ftp://ftp.ncbi.nlm.nih.gov/blast/db/) using WU-BLAST (BlastX) [31] with the following parameters: filter seg, W 6, WINK 6, nogap. Sequences that aligned to microbial references using either cross_match or WU-BLAST were confirmed by WU-BLAST alignment to the larger NT database. Virus alignments were then manually evaluated, and ambiguous alignments were removed. The same protocol was used for the analysis of the 75-mer data, except a minscore of 50 was used in the cross_match alignments. Detailed sequence statistics are presented in Figure S5. Figure S6 shows the number of virus sequences found with cross_match and BlastX, without scaling.

Viral sequences were assembled into contigs using Tigra (Chen L and Weinstock G, unpublished).

## Supporting Information

Figure S1 **General statistics on the number of sequences generated.** The mean, minimum, maximum, and median numbers of reads for each group of subjects are presented in the table. The distributions of the number of sequences generated for each subject group are plotted below.
(DOC)

Figure S2 **Lower Ct values (indicative of more virus) correlate with higher numbers of viral sequences.** The average Ct value (red squares) and the number of sequence reads generated (blue bars) are graphed for samples that were positive by both real-time PCR and sequencing for (A) adenovirus, (B) enterovirus, and (C) bocavirus and parechovirus.
(TIF)

Figure S3 **Samples with additional sequences generated to assess detection of rare sequences.**
(DOC)

Figure S4 **Accession numbers for sequence data sets.**
(DOC)

Figure S5 **Detailed sequence statistics for each sample.**
(DOC)

Figure S6 **Virus read counts for each sample.** The numbers represent all alignments from cross_match and BlastX, not scaled to 3 million reads per sample. Only viruses with ≥1 read per 3 million sequences were used in comparisons between febrile and afebrile groups, so more viruses are listed here than in the text describing those comparisons.
(DOC)
