# Supplementary figures and images for: Sequence Analysis of the Human Virome in Febrile and Afebrile Children
Source: PLoS One. 2012 Jun 13;7(6):e27735. doi: 10.1371/journal.pone.0027735 (PMC3374612; doi:10.1371/journal.pone.0027735)

Figure S1.

|  | Mean | Min | Max | Median |
| --- | --- | --- | --- | --- |
| NP Afebrile | 4,737,194 | 1,829,088 | 21,508,050 | 4,333,312 |
| NP Febrile | 4,626,180 | 1,761,070 | 21,924,556 | 4,504,803 |
| Plasma Afebrile | 3,178,984 | 882,022 | 6,482,352 | 2,436,239 |
| Plasma Febrile | 5,298,457 | 2,887,372 | 7,874,064 | 5,044,927 |
| All samples | 4,588,262 | 882,022 | 21,924,556 | 4,377,952 |


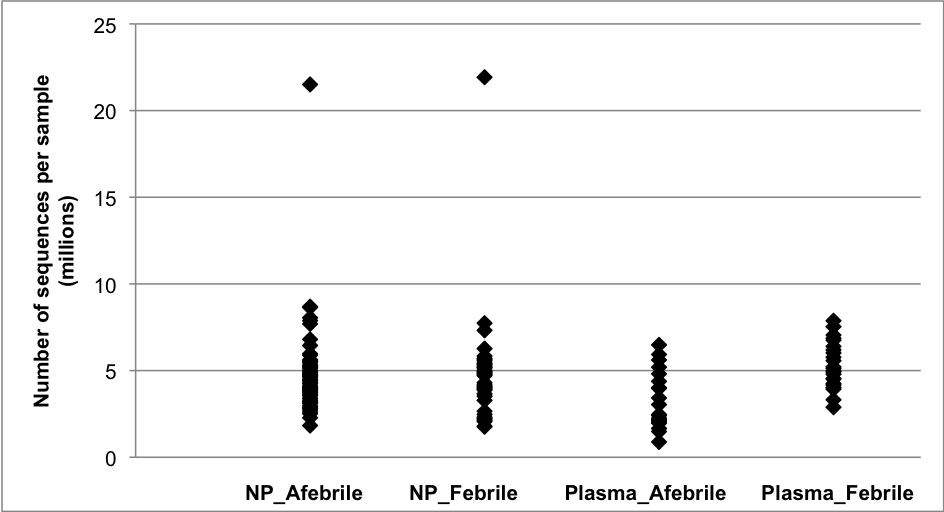

Supplement: Figure S1 — General statistics on the number of sequences generated. The mean, minimum, maximum, and median numbers of reads for each group of subjects are presented in the table. The distributions of the number of sequences generated for each subject group are plotted below. (DOC) [file pone.0027735.s001.doc]

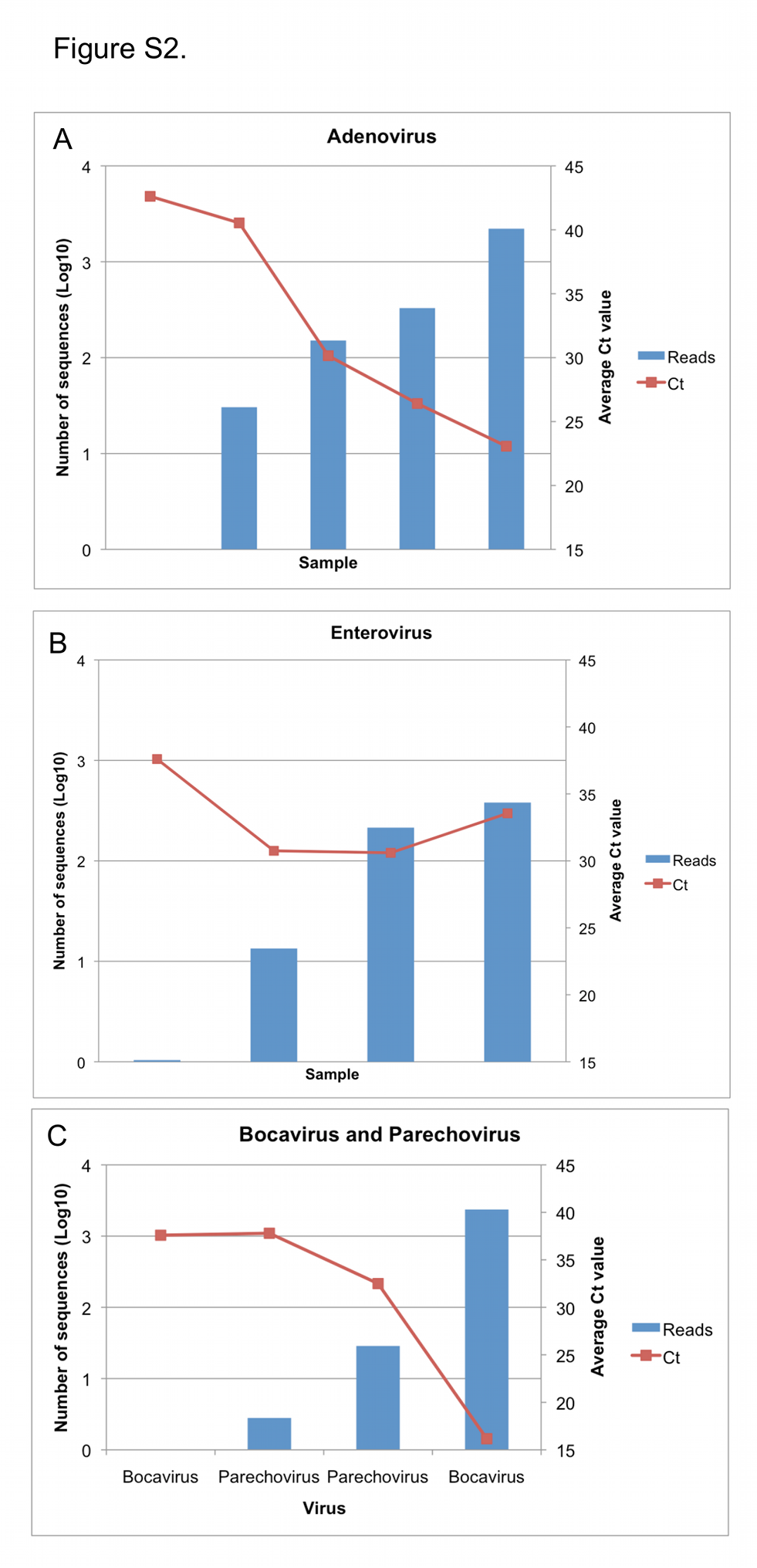

Supplement: Figure S2 — Lower Ct values (indicative of more virus) correlate with higher numbers of viral sequences. The average Ct value (red squares) and the number of sequence reads generated (blue bars) are graphed for samples that were positive by both real-time PCR and sequencing for (A) adenovirus, (B) enterovirus, and (C) bocavirus and parechovirus. (TIF) [file pone.0027735.s002.tif]
