# Supplementary material for: Sequence Analysis of the Human Virome in Febrile and Afebrile Children
Source: PLoS One. 2012 Jun 13;7(6):e27735. doi: 10.1371/journal.pone.0027735 (PMC3374612; doi:10.1371/journal.pone.0027735)
Supplement: Figure S3 — Samples with additional sequences generated to assess detection of rare sequences. (DOC) [file pone.0027735.s003.doc]

Figure S3. Samples with additional sequences generated to assess detection of rare sequences

| SID | TID | Additional sequences | Sequencing platform | Comments |
| --- | --- | --- | --- | --- |
| 9007 | 567 | 21,635,318 | GAIIX |  |
| 9013 | 573 | 51,625,900 | GAIIX and HiSeq |  |
| 9021 | 895 | 52,479,806 | GAIIX and Hiseq |  |
| 9021 | 581 | 21,553,320 | GAIIX |  |
| 9031 | 591 | 21,234,458 | GAIIX |  |
| 9105 | 663 | 4,248,418 | GAIIX | NP sample from a subject with respiratory symptoms used for Bocavirus sequencing |
