# Supplementary material for: Sequence Analysis of the Human Virome in Febrile and Afebrile Children
Source: PLoS One. 2012 Jun 13;7(6):e27735. doi: 10.1371/journal.pone.0027735 (PMC3374612; doi:10.1371/journal.pone.0027735)
Supplement: Figure S4 — Accession numbers for sequence data sets. (DOC) [file pone.0027735.s004.doc]

Figure S4. Accession numbers for sequence data sets.

| **Subject ID** | **Sample/Tube ID** | **Accession ID** | **Accession ID** | **Accession ID** | **Accession ID** |
| --- | --- | --- | --- | --- | --- |
| 9006 | 566 | SRR057873 |  |  |  |
| 9007 | 567 | SRR057890 | SRR316295 |  |  |
| 9008 | 568 | SRR057888 |  |  |  |
| 9009 | 569 | SRR057895 |  |  |  |
| 9011 | 571 | SRR056894 |  |  |  |
| 9012 | 572 | SRR057881 |  |  |  |
| 9013 | 573 | SRR057884 | SRR316298 |  |  |
| 9014 | 574 | SRR057872 |  |  |  |
| 9015 | 575 | SRR056887 |  |  |  |
| 9016 | 576 | SRR057893 |  |  |  |
| 9111 | 669 | SRR316218 | SRR316217 | SRR316231 | SRR316238 |
| 9112 | 670 | SRR316216 | SRR316232 | SRR316239 | SRR316268 |
| 9006 | 859 | SRR057860 | SRR057957 |  |  |
| 9007 | 840 | SRR057961 | SRR057858 |  |  |
| 9008 | 851 | SRR057960 | SRR057863 |  |  |
| 9011 | 869 | SRR057849 | SRR057966 |  |  |
| 9012 | 873 | SRR057967 | SRR057853 |  |  |
| 9014 | 878 | SRR057861 | SRR057964 |  |  |
| 9015 | 881 | SRR057963 | SRR057852 |  |  |
| 9016 | 883 | SRR057958 | SRR057865 |  |  |
| 9017 | 886 | SRR057962 | SRR057864 |  |  |
| 9019 | 579 | SRR057782 |  |  |  |
| 9019 | 892 | SRR057965 | SRR057854 |  |  |
| 9021 | 581 | SRR058029 |  |  |  |
| 9021 | 895 | SRR057938 | SRR057831 |  |  |
| 9022 | 582 | SRR057774 |  |  |  |
| 9022 | 898 | SRR057952 | SRR057824 |  |  |
| 9023 | 583 | SRR058054 |  |  |  |
| 9023 | 901 | SRR057949 | SRR057825 |  |  |
| 9025 | 585 | SRR057780 |  |  |  |
| 9025 | 906 | SRR057939 | SRR057832 |  |  |
| 9029 | 589 | SRR057976 | SRR057869 |  |  |
| 9029 | 915 | SRR057900 |  |  |  |
| 9030 | 590 | SRR057992 |  |  |  |
| 9030 | 918 | SRR057915 |  |  |  |
| 9031 | 591 | SRR057998 |  |  |  |
| 9031 | 921 | SRR057913 |  |  |  |
| 9033 | 593 | SRR057993 |  |  |  |
| 9034 | 594 | SRR057997 |  |  |  |
| 9034 | 928 | SRR057948 | SRR057830 |  |  |
| 9037 | 597 | SRR057999 |  |  |  |
| 9037 | 930 | SRR057827 | SRR057954 |  |  |
| 9040 | 600 | SRR057977 | SRR057866 |  |  |
| 9040 | 937 | SRR057940 | SRR057833 |  |  |
| 9042 | 602 | SRR057996 |  |  |  |
| 9044 | 604 | SRR058013 |  |  |  |
| 9044 | 943 | SRR057951 | SRR057823 |  |  |
| 9045 | 605 | SRR058018 |  |  |  |
| 9045 | 946 | SRR057956 | SRR057828 |  |  |
| 9047 | 800 | SRR058016 |  |  |  |
| 9047 | 952 | SRR057947 | SRR057834 |  |  |
| 9050 | 609 | SRR058008 |  |  |  |
| 9050 | 960 | SRR057936 | SRR057842 |  |  |
| 9051 | 610 | SRR058017 |  |  |  |
| 9051 | 963 | SRR057942 | SRR057836 |  |  |
| 9054 | 613 | SRR057991 |  |  |  |
| 9054 | 970 | SRR057946 | SRR057841 |  |  |
| 9055 | 614 | SRR057987 |  |  |  |
| 9055 | 973 | SRR057945 | SRR057838 |  |  |
| 9056 | 615 | SRR058002 |  |  |  |
| 9056 | 976 | SRR057899 |  |  |  |
| 9057 | 616 | SRR058004 |  |  |  |
| 9057 | 979 | SRR057937 | SRR057843 |  |  |
| 9059 | 618 | SRR057985 |  |  |  |
| 9059 | 996 | SRR057880 |  |  |  |
| 9060 | 619 | SRR057995 |  |  |  |
| 9060 | 1352 | SRR057876 |  |  |  |
| 9061 | 620 | SRR057988 |  |  |  |
| 9061 | 999 | SRR057879 |  |  |  |
| 9062 | 621 | SRR057989 |  |  |  |
| 9062 | 1002 | SRR057891 |  |  |  |
| 9063 | 622 | SRR058009 |  |  |  |
| 9063 | 1004 | SRR057874 |  |  |  |
| 9065 | 624 | SRR058007 |  |  |  |
| 9066 | 625 | SRR057994 |  |  |  |
| 9066 | 1013 | SRR057877 |  |  |  |
| 9067 | 626 | SRR058005 |  |  |  |
| 9070 | 629 | SRR058000 |  |  |  |
| 9071 | 630 | SRR058003 |  |  |  |
| 9072 | 631 | SRR057986 |  |  |  |
| 9072 | 1027 | SRR057878 |  |  |  |
| 9075 | 634 | SRR057983 |  |  |  |
| 9075 | 1036 | SRR057885 |  |  |  |
| 9076 | 635 | SRR057984 |  |  |  |
| 9076 | 1039 | SRR057896 |  |  |  |
| 9080 | 639 | SRR057797 |  |  |  |
| 9080 | 1050 | SRR057882 |  |  |  |
| 9081 | 640 | SRR057798 |  |  |  |
| 9081 | 1053 | SRR057886 |  |  |  |
| 9082 | 641 | SRR057805 |  |  |  |
| 9083 | 642 | SRR057914 |  |  |  |
| 9084 | 643 | SRR057816 |  |  |  |
| 9085 | 644 | SRR057793 |  |  |  |
| 9085 | 1065 | SRR057892 |  |  |  |
| 9086 | 645 | SRR057802 |  |  |  |
| 9087 | 646 | SRR057795 |  |  |  |
| 9087 | 1071 | SRR057875 |  |  |  |
| 9088 | 647 | SRR057804 |  |  |  |
| 9089 | 648 | SRR057794 |  |  |  |
| 9090 | 649 | SRR057810 |  |  |  |
| 9090 | 1078 | SRR057897 |  |  |  |
| 9091 | 650 | SRR057905 |  |  |  |
| 9091 | 1081 | SRR057898 |  |  |  |
| 9093 | 652 | SRR057796 |  |  |  |
| 9093 | 1087 | SRR057912 |  |  |  |
| 9094 | 653 | SRR057777 |  |  |  |
| 9095 | 799 | SRR057789 |  |  |  |
| 9096 | 654 | SRR057772 |  |  |  |
| 9097 | 655 | SRR057781 |  |  |  |
| 9098 | 656 | SRR057775 |  |  |  |
| 9099 | 657 | SRR057784 |  |  |  |
| 9100 | 658 | SRR057820 |  |  |  |
| 9101 | 659 | SRR058063 |  |  |  |
| 9102 | 660 | SRR057910 |  |  |  |
| 9103 | 661 | SRR058053 |  |  |  |
| 9105 | 663 | SRR058061 |  |  |  |
| 9106 | 664 | SRR058031 |  |  |  |
| 9107 | 665 | SRR058056 |  |  |  |
| 9109 | 667 | SRR058026 |  |  |  |
| 9110 | 668 | SRR057972 | SRR057857 |  |  |
| 9113 | 671 | SRR057790 |  |  |  |
| 9114 | 672 | SRR057783 |  |  |  |
| 9115 | 673 | SRR057785 |  |  |  |
| 9116 | 674 | SRR058052 |  |  |  |
| 9117 | 675 | SRR057908 |  |  |  |
| 9118 | 676 | SRR057923 |  |  |  |
| 9119 | 677 | SRR057922 |  |  |  |
| 9121 | 679 | SRR057907 |  |  |  |
| 9122 | 680 | SRR057920 |  |  |  |
| 9123 | 681 | SRR057909 |  |  |  |
| 9125 | 683 | SRR057786 |  |  |  |
| 9126 | 684 | SRR057918 |  |  |  |
| 9127 | 685 | SRR057901 |  |  |  |
| 9128 | 686 | SRR057788 |  |  |  |
| 9129 | 687 | SRR057973 | SRR057848 |  |  |
| 9130 | 688 | SRR057974 | SRR057839 |  |  |
| 9131 | 689 | SRR058044 |  |  |  |
| 9132 | 690 | SRR058059 |  |  |  |
| 9133 | 691 | SRR058032 |  |  |  |
| 9134 | 692 | SRR058019 |  |  |  |
| 9135 | 693 | SRR058041 |  |  |  |
| 9136 | 694 | SRR058033 |  |  |  |
| 9137 | 695 | SRR058022 |  |  |  |
| 9138 | 696 | SRR058034 |  |  |  |
| 9139 | 697 | SRR058035 |  |  |  |
| 9142 | 699 | SRR057929 |  |  |  |
| 9144 | 701 | SRR057813 |  |  |  |
| 9145 | 702 | SRR057807 |  |  |  |
| 9146 | 703 | SRR057812 |  |  |  |
| 9147 | 704 | SRR057806 |  |  |  |
| 9149 | 706 | SRR057791 |  |  |  |
| 9150 | 480 | SRR057792 |  |  |  |
| 9151 | 484 | SRR057809 |  |  |  |
| 9152 | 707 | SRR057799 |  |  |  |
| 9153 | 708 | SRR057801 |  |  |  |
| 9154 | 709 | SRR057800 |  |  |  |
| 9155 | 710 | SRR057803 |  |  |  |
| 9156 | 711 | SRR057979 | SRR057855 |  |  |
| 9157 | 712 | SRR057969 | SRR057844 |  |  |
| 9159 | 713 | SRR057980 | SRR057856 |  |  |
| 9160 | 714 | SRR057971 | SRR057847 |  |  |
| 9161 | 715 | SRR057978 | SRR057851 |  |  |
| 9162 | 716 | SRR057982 | SRR057846 |  |  |
| 9163 | 717 | SRR057975 | SRR057845 |  |  |
| 9166 | 720 | SRR057822 |  |  |  |
| 9168 | 722 | SRR058047 |  |  |  |
| 9170 | 724 | SRR058046 |  |  |  |
| 9171 | 725 | SRR058027 |  |  |  |
| 9172 | 726 | SRR058048 |  |  |  |
| 9179 | 727 | SRR058045 |  |  |  |
| 9182 | 729 | SRR058020 |  |  |  |
| 9184 | 730 | SRR058058 | SRR057926 |  |  |
| 9187 | 731 | SRR058055 | SRR057925 |  |  |
| 9193 | 732 | SRR058057 | SRR057906 |  |  |
| 9194 | 733 | SRR058040 | SRR057917 |  |  |
| 9195 | 734 | SRR058042 | SRR057924 |  |  |
| 9204 | 736 | SRR058028 | SRR057902 |  |  |
