# Supplementary material for: Sequence Analysis of the Human Virome in Febrile and Afebrile Children
Source: PLoS One. 2012 Jun 13;7(6):e27735. doi: 10.1371/journal.pone.0027735 (PMC3374612; doi:10.1371/journal.pone.0027735)
Supplement: Figure S5 — Detailed sequence statistics for each sample. (DOC) [file pone.0027735.s005.doc]

Figure S5. Detailed sequence statistics for each sample

| Subject ID | Tube ID | Total sequences | Unique sequences | Pass prescreen | Total human | Pass human screen | Fever status | Sample source |
| --- | --- | --- | --- | --- | --- | --- | --- | --- |
| 9050 | 609 | 2,277,482 | 2,231,398 | 1,823,685 | 1,293,232 | 530,453 | A | NP |
| 9051 | 610 | 2,806,712 | 2,717,642 | 2,194,508 | 1,420,269 | 774,239 | A | NP |
| 9054 | 613 | 2,978,682 | 2,774,850 | 2,361,078 | 1,621,290 | 739,788 | A | NP |
| 9055 | 614 | 3,262,768 | 3,041,706 | 2,403,081 | 1,630,711 | 772,370 | A | NP |
| 9056 | 615 | 3,211,228 | 3,094,546 | 2,442,153 | 1,679,926 | 762,227 | A | NP |
| 9057 | 616 | 5,968,574 | 5,676,856 | 4,689,261 | 3,139,756 | 1,549,505 | A | NP |
| 9059 | 618 | 3,174,954 | 3,135,047 | 2,476,338 | 1,682,018 | 794,320 | A | NP |
| 9060 | 619 | 3,910,488 | 3,725,541 | 3,028,368 | 1,455,956 | 1,572,412 | A | NP |
| 9061 | 620 | 3,410,606 | 3,284,470 | 2,799,636 | 1,882,896 | 916,740 | A | NP |
| 9062 | 621 | 4,059,596 | 3,978,109 | 3,063,166 | 1,929,834 | 1,133,332 | A | NP |
| 9063 | 622 | 2,895,152 | 2,871,660 | 2,247,886 | 1,398,493 | 849,393 | A | NP |
| 9066 | 625 | 5,179,156 | 4,654,873 | 4,105,324 | 2,567,887 | 1,537,437 | A | NP |
| 9067 | 626 | 3,117,020 | 3,085,603 | 2,567,756 | 1,764,135 | 803,621 | A | NP |
| 9071 | 630 | 4,107,738 | 4,019,522 | 3,315,816 | 2,193,795 | 1,122,021 | A | NP |
| 9072 | 631 | 1,829,088 | 1,726,178 | 1,465,189 | 977,159 | 360,481 | A | NP |
| 9075 | 634 | 2,509,926 | 2,368,957 | 2,011,295 | 1,484,825 | 526,470 | A | NP |
| 9076 | 635 | 3,158,720 | 3,036,087 | 2,545,711 | 1,788,789 | 756,922 | A | NP |
| 9080 | 639 | 4,655,356 | 3,904,991 | 3,677,784 | 1,453,731 | 2,224,053 | A | NP |
| 9081 | 640 | 4,438,854 | 4,166,691 | 3,774,754 | 2,227,184 | 1,547,570 | A | NP |
| 9082 | 641 | 3,765,660 | 3,650,293 | 3,130,837 | 1,924,313 | 1,206,524 | A | NP |
| 9083 | 642 | 5,624,946 | 2,845,498 | 2,474,082 | 472,461 | 2,001,621 | A | NP |
| 9085 | 644 | 5,563,376 | 5,521,429 | 4,532,144 | 2,510,542 | 2,021,602 | A | NP |
| 9087 | 646 | 4,333,312 | 4,239,868 | 3,640,169 | 2,087,573 | 1,552,596 | A | NP |
| 9088 | 647 | 4,495,552 | 4,146,421 | 3,568,753 | 1,624,531 | 1,944,222 | A | NP |
| 9090 | 649 | 4,749,996 | 4,187,708 | 3,748,933 | 1,980,496 | 1,768,437 | A | NP |
| 9091 | 650 | 5,155,176 | 2,552,759 | 2,354,291 | 1,045,495 | 1,308,796 | A | NP |
| 9093 | 652 | 4,038,322 | 3,909,636 | 3,250,146 | 1,966,685 | 1,283,461 | A | NP |
| 9094 | 653 | 4,704,500 | 4,652,350 | 3,630,137 | 1,818,644 | 1,811,493 | A | NP |
| 9095 | 799 | 4,652,670 | 4,617,408 | 3,701,183 | 1,931,448 | 1,769,735 | A | NP |
| 9096 | 654 | 4,232,068 | 3,816,067 | 3,390,271 | 1,980,241 | 1,410,030 | A | NP |
| 9097 | 655 | 5,279,788 | 5,161,545 | 4,195,514 | 2,331,011 | 1,864,503 | A | NP |
| 9098 | 656 | 5,331,488 | 5,182,349 | 4,318,789 | 2,300,394 | 2,018,395 | A | NP |
| 9099 | 657 | 5,885,026 | 5,539,891 | 4,765,645 | 2,243,326 | 2,522,319 | A | NP |
| 9101 | 659 | 5,265,184 | 5,106,605 | 4,313,365 | 2,059,259 | 2,254,106 | A | NP |
| 9102 | 660 | 3,966,336 | 3,876,475 | 3,268,174 | 1,778,434 | 1,489,740 | A | NP |
| 9103 | 661 | 3,594,026 | 3,473,349 | 2,886,159 | 1,886,142 | 1,000,017 | A | NP |
| 9106 | 664 | 4,411,106 | 4,249,303 | 3,672,224 | 2,374,375 | 1,297,849 | A | NP |
| 9107 | 665 | 2,836,502 | 2,783,753 | 2,222,329 | 1,623,416 | 598,913 | A | NP |
| 9110 | 668 | 5,522,312 | 5,320,399 | 4,507,646 | 2,647,340 | 1,860,306 | A | NP |
| 9111 | 669 | 21,508,050 | 15,973,545 | 14,764,512 | 6,133,329 | 8,631,183 | A | NP |
| 9113 | 671 | 4,272,676 | 4,229,135 | 3,760,726 | 1,993,217 | 1,767,509 | A | NP |
| 9114 | 672 | 3,846,708 | 3,599,786 | 3,288,882 | 1,849,843 | 1,439,039 | A | NP |
| 9115 | 673 | 6,445,324 | 5,931,921 | 5,471,666 | 3,024,394 | 2,447,272 | A | NP |
| 9116 | 674 | 2,725,570 | 2,690,019 | 2,413,001 | 1,450,206 | 962,795 | A | NP |
| 9117 | 675 | 4,788,296 | 4,606,749 | 4,021,217 | 2,088,586 | 1,932,631 | A | NP |
| 9118 | 676 | 5,010,234 | 4,917,885 | 4,053,605 | 2,192,654 | 1,860,951 | A | NP |
| 9121 | 679 | 4,042,620 | 3,768,811 | 3,362,433 | 1,776,546 | 1,585,887 | A | NP |
| 9122 | 680 | 4,001,354 | 3,649,312 | 3,260,750 | 1,753,011 | 1,507,739 | A | NP |
| 9123 | 681 | 3,737,092 | 3,599,463 | 3,157,830 | 1,769,088 | 1,388,742 | A | NP |
| 9125 | 683 | 6,450,488 | 6,221,735 | 5,638,257 | 3,398,920 | 2,239,337 | A | NP |
| 9127 | 685 | 3,802,704 | 3,669,253 | 3,257,977 | 1,861,691 | 1,396,286 | A | NP |
| 9128 | 686 | 6,800,054 | 6,320,589 | 5,724,406 | 2,817,901 | 2,906,505 | A | NP |
| 9129 | 687 | 5,902,950 | 5,768,995 | 4,994,189 | 3,121,634 | 1,872,555 | A | NP |
| 9130 | 688 | 5,233,120 | 5,135,111 | 4,371,322 | 2,681,790 | 1,689,532 | A | NP |
| 9131 | 689 | 4,652,694 | 4,388,438 | 3,641,169 | 2,627,990 | 1,013,179 | A | NP |
| 9132 | 690 | 3,741,378 | 3,577,146 | 3,090,289 | 2,099,386 | 990,903 | A | NP |
| 9133 | 691 | 2,612,508 | 2,575,919 | 1,917,259 | 1,267,578 | 649,681 | A | NP |
| 9134 | 692 | 3,107,476 | 2,996,609 | 2,565,274 | 1,812,354 | 752,920 | A | NP |
| 9135 | 693 | 3,886,790 | 3,669,489 | 3,133,145 | 1,883,621 | 1,249,524 | A | NP |
| 9136 | 694 | 3,365,254 | 3,250,595 | 2,780,183 | 1,754,773 | 1,025,410 | A | NP |
| 9137 | 695 | 3,726,080 | 3,598,538 | 2,989,682 | 1,716,540 | 1,273,142 | A | NP |
| 9142 | 699 | 4,611,326 | 4,256,642 | 3,771,942 | 1,817,353 | 1,954,589 | A | NP |
| 9144 | 701 | 4,851,178 | 4,566,339 | 3,664,435 | 1,790,105 | 1,874,330 | A | NP |
| 9145 | 702 | 4,097,388 | 2,625,777 | 2,215,597 | 1,301,501 | 914,096 | A | NP |
| 9146 | 703 | 5,130,322 | 4,785,961 | 4,116,677 | 2,515,284 | 1,601,393 | A | NP |
| 9147 | 704 | 4,249,542 | 4,062,893 | 3,409,986 | 1,988,945 | 1,421,041 | A | NP |
| 9149 | 706 | 4,025,060 | 3,883,967 | 3,229,284 | 1,920,688 | 1,308,596 | A | NP |
| 9150 | 480 | 4,737,722 | 4,587,633 | 3,818,961 | 2,209,392 | 1,609,569 | A | NP |
| 9151 | 484 | 3,662,720 | 3,482,958 | 2,825,820 | 1,750,856 | 1,074,964 | A | NP |
| 9152 | 707 | 4,922,058 | 4,717,150 | 3,041,411 | 1,577,657 | 1,463,754 | A | NP |
| 9159 | 713 | 4,936,142 | 4,648,652 | 4,061,993 | 2,336,093 | 1,725,900 | A | NP |
| 9162 | 716 | 3,900,656 | 3,803,444 | 3,216,484 | 1,919,383 | 1,297,101 | A | NP |
| 9163 | 717 | 5,459,962 | 5,414,558 | 4,303,732 | 2,579,663 | 1,724,069 | A | NP |
| 9166 | 720 | 7,694,534 | 7,508,675 | 6,446,878 | 3,246,353 | 3,200,525 | A | NP |
| 9168 | 722 | 5,134,270 | 5,070,588 | 4,320,433 | 2,524,194 | 1,796,239 | A | NP |
| 9179 | 727 | 5,474,770 | 5,253,638 | 4,470,741 | 2,313,934 | 2,156,807 | A | NP |
| 9182 | 729 | 3,540,890 | 3,439,030 | 2,858,204 | 1,586,336 | 1,271,868 | A | NP |
| 9184 | 730 | 8,641,612 | 8,061,167 | 7,086,574 | 3,720,094 | 3,366,480 | A | NP |
| 9187 | 731 | 8,049,360 | 7,632,476 | 6,879,848 | 3,852,192 | 3,027,656 | A | NP |
| 9194 | 733 | 7,884,474 | 7,660,025 | 6,607,448 | 3,760,722 | 2,846,726 | A | NP |
| 9195 | 734 | 8,689,934 | 7,913,027 | 7,092,719 | 3,600,033 | 3,492,686 | A | NP |
| 9006 | 566 | 1,761,070 | 1,643,853 | 1,309,061 | 604,880 | 704,181 | F | NP |
| 9007 | 567 | 2,219,532 | 2,121,288 | 1,615,838 | 693,215 | 922,623 | F | NP |
| 9008 | 568 | 1,788,670 | 1,736,238 | 1,399,432 | 731,593 | 667,839 | F | NP |
| 9009 | 569 | 2,139,402 | 2,044,639 | 1,533,823 | 629,635 | 904,188 | F | NP |
| 9011 | 571 | 2,170,256 | 2,080,052 | 1,492,036 | 603,644 | 888,392 | F | NP |
| 9012 | 572 | 2,653,326 | 2,587,264 | 1,926,762 | 684,659 | 1,242,103 | F | NP |
| 9013 | 573 | 2,293,754 | 2,224,632 | 1,715,510 | 714,427 | 1,001,083 | F | NP |
| 9014 | 574 | 1,765,684 | 1,719,406 | 1,352,321 | 657,471 | 694,850 | F | NP |
| 9015 | 575 | 2,061,242 | 1,973,395 | 1,465,910 | 502,000 | 963,910 | F | NP |
| 9016 | 576 | 2,313,842 | 2,287,968 | 1,844,054 | 911,454 | 932,600 | F | NP |
| 9019 | 579 | 5,422,450 | 4,994,242 | 4,533,766 | 2,019,303 | 2,514,463 | F | NP |
| 9021 | 581 | 4,144,016 | 3,905,700 | 3,486,687 | 1,815,330 | 1,671,357 | F | NP |
| 9022 | 582 | 5,172,110 | 4,654,790 | 4,171,071 | 2,291,868 | 1,879,203 | F | NP |
| 9023 | 583 | 3,961,922 | 3,888,728 | 3,190,391 | 1,586,763 | 1,603,628 | F | NP |
| 9025 | 585 | 3,903,386 | 3,801,765 | 3,383,965 | 1,791,455 | 1,592,510 | F | NP |
| 9029 | 589 | 5,228,766 | 4,938,307 | 3,841,867 | 1,979,821 | 1,862,046 | F | NP |
| 9030 | 590 | 4,298,162 | 3,714,293 | 2,744,392 | 1,147,824 | 1,596,568 | F | NP |
| 9031 | 591 | 3,658,940 | 3,488,456 | 2,809,854 | 1,961,945 | 847,909 | F | NP |
| 9033 | 593 | 5,630,644 | 5,560,248 | 4,428,441 | 2,794,641 | 1,633,800 | F | NP |
| 9034 | 594 | 5,571,842 | 5,478,264 | 4,231,677 | 2,758,299 | 1,473,378 | F | NP |
| 9037 | 597 | 3,657,508 | 3,019,146 | 2,119,306 | 1,115,392 | 1,003,914 | F | NP |
| 9040 | 600 | 5,146,092 | 5,010,235 | 3,709,682 | 1,628,872 | 2,080,810 | F | NP |
| 9042 | 602 | 3,518,434 | 3,487,223 | 2,855,876 | 1,803,072 | 1,052,804 | F | NP |
| 9044 | 604 | 5,324,936 | 5,256,109 | 4,399,717 | 2,549,454 | 1,850,263 | F | NP |
| 9045 | 605 | 5,008,052 | 4,937,231 | 3,805,192 | 2,359,802 | 1,445,390 | F | NP |
| 9047 | 800 | 2,467,258 | 2,049,545 | 1,715,004 | 741,470 | 973,534 | F | NP |
| 9065 | 624 | 4,029,572 | 3,793,171 | 3,177,889 | 2,131,708 | 1,046,181 | F | NP |
| 9070 | 629 | 4,093,396 | 4,002,027 | 3,367,460 | 2,251,443 | 1,116,017 | F | NP |
| 9084 | 643 | 5,190,410 | 5,078,401 | 4,437,048 | 2,554,933 | 1,859,975 | F | NP |
| 9086 | 645 | 5,432,592 | 5,147,116 | 4,541,609 | 2,194,391 | 2,347,218 | F | NP |
| 9089 | 648 | 4,777,044 | 4,483,804 | 3,872,259 | 2,125,011 | 1,747,248 | F | NP |
| 9100 | 658 | 4,892,214 | 4,510,098 | 4,064,265 | 2,242,335 | 1,821,930 | F | NP |
| 9109 | 667 | 4,814,112 | 4,511,862 | 3,383,738 | 2,285,435 | 1,098,303 | F | NP |
| 9112 | 670 | 21,924,556 | 16,516,635 | 15,035,251 | 6,652,533 | 8,382,718 | F | NP |
| 9119 | 677 | 4,171,830 | 4,115,955 | 3,352,464 | 1,763,072 | 1,589,392 | F | NP |
| 9126 | 684 | 4,036,216 | 3,717,513 | 3,384,959 | 1,803,028 | 1,581,931 | F | NP |
| 9138 | 696 | 3,282,772 | 3,136,778 | 2,568,990 | 1,563,154 | 1,005,836 | F | NP |
| 9139 | 697 | 3,872,660 | 3,704,073 | 2,409,239 | 1,098,238 | 1,311,001 | F | NP |
| 9153 | 708 | 4,963,444 | 4,303,688 | 3,559,396 | 2,049,199 | 1,510,197 | F | NP |
| 9154 | 709 | 5,703,822 | 5,516,395 | 4,504,756 | 2,696,353 | 1,808,403 | F | NP |
| 9155 | 710 | 6,267,370 | 5,790,886 | 4,739,466 | 2,711,084 | 2,028,382 | F | NP |
| 9156 | 711 | 4,870,758 | 4,672,565 | 3,896,289 | 2,297,498 | 1,598,791 | F | NP |
| 9157 | 712 | 5,172,860 | 5,083,242 | 4,211,548 | 2,253,016 | 1,958,532 | F | NP |
| 9160 | 714 | 5,403,782 | 4,984,049 | 4,181,142 | 2,267,695 | 1,913,447 | F | NP |
| 9161 | 715 | 4,158,864 | 3,891,449 | 3,136,642 | 1,436,783 | 1,699,859 | F | NP |
| 9170 | 724 | 5,838,328 | 5,477,209 | 4,816,411 | 2,515,610 | 2,300,801 | F | NP |
| 9171 | 725 | 5,363,598 | 4,552,679 | 4,148,614 | 2,328,002 | 1,820,612 | F | NP |
| 9172 | 726 | 4,711,444 | 4,544,181 | 3,758,253 | 2,241,944 | 1,516,309 | F | NP |
| 9193 | 732 | 7,321,398 | 6,541,350 | 5,884,467 | 2,792,690 | 3,091,777 | F | NP |
| 9204 | 736 | 7,734,698 | 6,850,741 | 6,194,764 | 3,059,000 | 3,135,764 | F | NP |
| 9050 | 960 | 4,377,952 | 3,172,436 | 3,058,575 | 1,365,138 | 1,693,437 | A | P |
| 9051 | 963 | 3,973,206 | 2,787,217 | 2,706,149 | 1,072,053 | 1,634,096 | A | P |
| 9054 | 970 | 5,929,446 | 5,210,355 | 4,338,998 | 2,161,102 | 2,177,896 | A | P |
| 9055 | 973 | 5,612,092 | 4,949,971 | 3,738,683 | 1,841,144 | 1,897,539 | A | P |
| 9056 | 976 | 4,810,034 | 4,376,730 | 3,172,399 | 149,161 | 3,023,238 | A | P |
| 9057 | 979 | 6,482,352 | 5,761,466 | 4,977,070 | 2,505,460 | 2,471,610 | A | P |
| 9059 | 996 | 1,665,074 | 1,546,155 | 1,315,288 | 720,490 | 594,798 | A | P |
| 9060 | 1352 | 882,022 | 802,660 | 662,615 | 358,169 | 304,446 | A | P |
| 9061 | 999 | 2,089,216 | 1,873,635 | 1,230,654 | 523,637 | 707,017 | A | P |
| 9062 | 1002 | 2,058,592 | 1,790,016 | 1,430,319 | 668,672 | 761,647 | A | P |
| 9063 | 1004 | 2,175,556 | 1,924,797 | 1,466,380 | 696,428 | 769,952 | A | P |
| 9066 | 1013 | 1,483,486 | 1,104,055 | 995,513 | 393,728 | 601,785 | A | P |
| 9072 | 1027 | 1,993,212 | 1,728,773 | 1,247,572 | 582,100 | 665,472 | A | P |
| 9075 | 1036 | 2,467,162 | 2,229,725 | 1,589,052 | 736,175 | 852,877 | A | P |
| 9076 | 1039 | 5,194,312 | 4,595,853 | 3,308,909 | 1,418,864 | 1,890,045 | A | P |
| 9080 | 1050 | 2,405,316 | 2,238,469 | 1,397,888 | 620,040 | 777,848 | A | P |
| 9081 | 1053 | 2,246,226 | 2,063,582 | 1,785,955 | 913,445 | 872,510 | A | P |
| 9085 | 1065 | 1,656,394 | 1,207,035 | 1,118,469 | 482,600 | 635,869 | A | P |
| 9087 | 1071 | 1,967,238 | 1,736,125 | 1,491,859 | 782,210 | 709,649 | A | P |
| 9090 | 1078 | 3,419,788 | 2,343,170 | 2,159,229 | 831,157 | 1,328,072 | A | P |
| 9091 | 1081 | 3,038,664 | 2,124,933 | 1,839,477 | 830,078 | 1,009,399 | A | P |
| 9093 | 1087 | 4,010,324 | 2,878,412 | 2,429,359 | 901,173 | 1,528,186 | A | P |
| 9006 | 859 | 4,250,120 | 3,200,255 | 2,926,810 | 1,509,522 | 1,417,288 | F | P |
| 9007 | 840 | 6,858,076 | 6,206,211 | 4,853,158 | 2,049,980 | 2,803,178 | F | P |
| 9008 | 851 | 4,792,380 | 3,914,295 | 3,253,523 | 1,596,733 | 1,656,790 | F | P |
| 9011 | 869 | 5,124,270 | 4,752,104 | 3,293,921 | 1,673,887 | 1,620,034 | F | P |
| 9012 | 873 | 7,532,674 | 7,178,973 | 4,542,225 | 2,373,968 | 2,168,257 | F | P |
| 9014 | 878 | 4,168,012 | 3,956,435 | 2,765,313 | 1,312,922 | 1,452,391 | F | P |
| 9015 | 881 | 4,541,462 | 4,373,129 | 3,254,515 | 1,582,860 | 1,671,655 | F | P |
| 9016 | 883 | 2,887,372 | 2,165,874 | 2,105,558 | 848,978 | 1,256,580 | F | P |
| 9017 | 886 | 4,525,910 | 4,362,243 | 3,315,797 | 1,794,450 | 1,521,347 | F | P |
| 9019 | 892 | 5,767,792 | 4,833,910 | 4,242,462 | 2,058,375 | 2,184,087 | F | P |
| 9021 | 895 | 4,965,584 | 4,911,588 | 3,215,583 | 1,580,966 | 1,634,617 | F | P |
| 9022 | 898 | 3,931,804 | 2,989,229 | 2,796,589 | 1,101,710 | 1,694,879 | F | P |
| 9023 | 901 | 4,501,996 | 4,363,546 | 3,676,777 | 1,649,376 | 2,027,401 | F | P |
| 9025 | 906 | 4,023,524 | 3,013,606 | 2,909,230 | 1,215,102 | 1,694,128 | F | P |
| 9028 | 912 | 3,314,922 | 2,495,357 | 2,295,468 | 991,218 | 1,304,250 | F | P |
| 9029 | 915 | 6,189,578 | 5,492,378 | 4,391,275 | 210,412 | 4,180,863 | F | P |
| 9030 | 918 | 6,017,228 | 5,468,781 | 4,367,436 | 991,927 | 3,375,509 | F | P |
| 9031 | 921 | 5,211,726 | 4,796,369 | 3,623,507 | 393,948 | 3,229,559 | F | P |
| 9034 | 928 | 6,755,548 | 6,406,491 | 5,541,498 | 2,190,812 | 3,350,686 | F | P |
| 9037 | 930 | 4,918,142 | 4,220,634 | 3,476,723 | 1,495,877 | 1,980,846 | F | P |
| 9040 | 937 | 5,571,666 | 4,956,423 | 4,421,905 | 2,296,042 | 2,125,863 | F | P |
| 9044 | 943 | 7,874,064 | 7,438,289 | 5,067,890 | 2,183,291 | 2,884,599 | F | P |
| 9045 | 946 | 7,044,716 | 6,849,549 | 4,942,696 | 2,072,173 | 2,870,523 | F | P |
| 9047 | 952 | 6,394,424 | 5,942,849 | 4,888,913 | 2,350,397 | 2,538,516 | F | P |
