# Supplementary material for: Sequence Analysis of the Human Virome in Febrile and Afebrile Children
Source: PLoS One. 2012 Jun 13;7(6):e27735. doi: 10.1371/journal.pone.0027735 (PMC3374612; doi:10.1371/journal.pone.0027735)
Supplement: Figure S6 — Virus read counts for each sample. The numbers represent all alignments from cross_match and BlastX, not scaled to 3 million reads per sample. Only viruses with ≥1 read per 3 million sequences were used in comparisons between febrile and afebrile groups, so more viruses are listed here than in the text describing those comparisons. (DOC) [file pone.0027735.s006.doc]

Figure S6. Virus read counts for each sample. The numbers represent all alignments from cross_match and BlastX, not scaled to 3 million reads per sample. Only viruses with > 1 read per 3 million sequences were used in comparisons between febrile and afebrile groups, so more viruses are listed here than in the text describing those comparisons.

Plasma – Febrile

| Subject ID | Tube ID | | | Astrovirus | | | Cytomegalovirus | | | Dependovirus | | | Enterovirus | | | Erythrovirus | Flaviviridae - HepG | Hepacivirus | Othohepadnavirus |
| --- | --- | --- | --- | --- | --- | --- | --- | --- | --- | --- | --- | --- | --- | --- | --- | --- | --- | --- | --- |
| 9006 | 859 | | | 0 | | | 0 | | | 0 | | | 0 | | | 0 | 0 | 0 | 0 |
| 9007 | 840 | | | 0 | | | 0 | | | 0 | | | 3 | | | 0 | 0 | 0 | 0 |
| 9008 | 851 | | | 0 | | | 0 | | | 0 | | | 342 | | | 0 | 0 | 0 | 0 |
| 9011 | 869 | | | 0 | | | 0 | | | 0 | | | 0 | | | 0 | 0 | 0 | 0 |
| 9012 | 873 | | | 0 | | | 0 | | | 0 | | | 0 | | | 0 | 0 | 0 | 0 |
| 9014 | 878 | | | 0 | | | 0 | | | 0 | | | 0 | | | 0 | 0 | 0 | 0 |
| 9015 | 881 | | | 0 | | | 0 | | | 0 | | | 576 | | | 0 | 0 | 0 | 0 |
| 9016 | 883 | | | 0 | | | 0 | | | 0 | | | 1 | | | 0 | 0 | 0 | 0 |
| 9017 | 886 | | | 0 | | | 0 | | | 0 | | | 0 | | | 2 | 0 | 0 | 0 |
| 9019 | 892 | | | 0 | | | 0 | | | 0 | | | 0 | | | 0 | 0 | 0 | 0 |
| 9021 | 895 | | | 0 | | | 0 | | | 9250 | | | 0 | | | 0 | 0 | 0 | 0 |
| 9022 | 898 | | | 0 | | | 0 | | | 0 | | | 2 | | | 0 | 0 | 0 | 0 |
| 9023 | 901 | | | 0 | | | 0 | | | 0 | | | 0 | | | 0 | 0 | 0 | 0 |
| 9025 | 906 | | | 0 | | | 0 | | | 0 | | | 18 | | | 0 | 0 | 0 | 0 |
| 9028 | 912 | | | 0 | | | 0 | | | 0 | | | 0 | | | 0 | 0 | 0 | 0 |
| 9029 | 915 | | | 0 | | | 0 | | | 0 | | | 0 | | | 0 | 0 | 0 | 4 |
| 9030 | 918 | | | 0 | | | 0 | | | 0 | | | 0 | | | 0 | 0 | 0 | 0 |
| 9031 | 921 | | | 0 | | | 0 | | | 0 | | | 49 | | | 0 | 0 | 0 | 0 |
| 9034 | 928 | | | 0 | | | 0 | | | 1 | | | 0 | | | 0 | 4 | 0 | 0 |
| 9037 | 930 | | | 0 | | | 0 | | | 0 | | | 0 | | | 0 | 0 | 1 | 0 |
| 9040 | 937 | | | 0 | | | 0 | | | 0 | | | 0 | | | 0 | 0 | 0 | 0 |
| 9044 | 943 | | | 0 | | | 2 | | | 0 | | | 0 | | | 0 | 0 | 0 | 0 |
| 9045 | 946 | | | 0 | | | 0 | | | 0 | | | 0 | | | 0 | 0 | 0 | 0 |
| 9047 | 952 | | | 376 | | | 0 | | | 0 | | | 0 | | | 0 | 0 | 0 | 0 |
| Subject ID | Tube ID | | Influenzavirus A | | | Mastadenovirus | | | Rhinovirus | | Roseolovirus | | | |  | | | | |
| 9006 | 859 | | 0 | | | 0 | | | 0 | | 0 | | | |  | | | | |
| 9007 | 840 | | 0 | | | 0 | | | 0 | | 0 | | | |  | | | | |
| 9008 | 851 | | 0 | | | 0 | | | 0 | | 0 | | | |  | | | | |
| 9011 | 869 | | 0 | | | 0 | | | 0 | | 0 | | | |  | | | | |
| 9012 | 873 | | 0 | | | 0 | | | 0 | | 0 | | | |  | | | | |
| 9014 | 878 | | 0 | | | 0 | | | 0 | | 42 | | | |  | | | | |
| 9015 | 881 | | 0 | | | 0 | | | 0 | | 0 | | | |  | | | | |
| 9016 | 883 | | 0 | | | 0 | | | 0 | | 0 | | | |  | | | | |
| 9017 | 886 | | 0 | | | 0 | | | 0 | | 4 | | | |  | | | | |
| 9019 | 892 | | 1 | | | 0 | | | 0 | | 0 | | | |  | | | | |
| 9021 | 895 | | 0 | | | 0 | | | 0 | | 0 | | | |  | | | | |
| 9022 | 898 | | 0 | | | 0 | | | 0 | | 8 | | | |  | | | | |
| 9023 | 901 | | 0 | | | 0 | | | 0 | | 80 | | | |  | | | | |
| 9025 | 906 | 0 | | | 0 | | | 0 | | | | 0 | |  | | | | | |
| 9028 | 912 | 0 | | | 0 | | | 0 | | | | 0 | |  | | | | | |
| 9029 | 915 | 0 | | | 0 | | | 0 | | | | 0 | |  | | | | | |
| 9030 | 918 | 0 | | | 0 | | | 0 | | | | 0 | |  | | | | | |
| 9031 | 921 | 0 | | | 0 | | | 0 | | | | 0 | |  | | | | | |
| 9034 | 928 | 0 | | | 0 | | | 0 | | | | 12 | |  | | | | | |
| 9037 | 930 | 0 | | | 0 | | | 0 | | | | 0 | |  | | | | | |
| 9040 | 937 | 0 | | | 0 | | | 0 | | | | 3 | |  | | | | | |
| 9044 | 943 | 0 | | | 0 | | | 0 | | | | 1117 | |  | | | | | |
| 9045 | 946 | 0 | | | 0 | | | 0 | | | | 1 | |  | | | | | |
| 9047 | 952 | 0 | | | 0 | | | 0 | | | | 0 | |  | | | | | |

Plasma – Afebrile

| Subject ID | Tube ID | Anellovirus |
| --- | --- | --- |
| 9050 | 960 | 11 |
| 9051 | 963 | 13 |
| 9054 | 970 | 212 |
| 9055 | 973 | 4 |
| 9056 | 976 | 17 |
| 9057 | 979 | 4 |
| 9059 | 996 | 0 |
| 9060 | 1352 | 18 |
| 9061 | 999 | 5 |
| 9062 | 1002 | 90 |
| 9063 | 1004 | 20 |
| 9066 | 1013 | 0 |
| 9072 | 1027 | 64 |
| 9075 | 1036 | 0 |
| 9076 | 1039 | 0 |
| 9080 | 1050 | 12 |
| 9081 | 1053 | 49 |
| 9085 | 1065 | 2 |
| 9087 | 1071 | 0 |
| 9090 | 1078 | 0 |
| 9091 | 1081 | 6 |
| 9093 | 1087 | 13 |

NP - Febrile

| Subject ID | Tube ID | Anellovirus | Astrovirus | Betapapillomavirus | Bocavirus | Coltivirus | Cytomegalovirus | Dependovirus | Enterovirus | Influenzavirus A | Mastadenovirus | Metapneumovirus | Morbillivirus |
| --- | --- | --- | --- | --- | --- | --- | --- | --- | --- | --- | --- | --- | --- |
| 9006 | 566 | 0 | 0 | 0 | 0 | 0 | 0 | 0 | 22 | 0 | 0 | 0 | 0 |
| 9007 | 567 | 0 | 0 | 0 | 0 | 0 | 0 | 0 | 0 | 0 | 17608 | 0 | 0 |
| 9008 | 568 | 0 | 0 | 0 | 0 | 0 | 0 | 0 | 0 | 0 | 0 | 0 | 0 |
| 9009 | 569 | 0 | 0 | 0 | 0 | 0 | 0 | 0 | 0 | 0 | 0 | 0 | 0 |
| 9011 | 571 | 3 | 0 | 0 | 0 | 0 | 0 | 0 | 2 | 0 | 22 | 0 | 0 |
| 9012 | 572 | 0 | 0 | 0 | 0 | 0 | 0 | 0 | 0 | 0 | 0 | 0 | 0 |
| 9013 | 573 | 62 | 0 | 0 | 4 | 0 | 0 | 0 | 0 | 0 | 2713 | 0 | 0 |
| 9014 | 574 | 1 | 0 | 0 | 0 | 0 | 0 | 0 | 0 | 0 | 0 | 0 | 0 |
| 9015 | 575 | 0 | 0 | 0 | 0 | 0 | 0 | 0 | 0 | 0 | 0 | 0 | 0 |
| 9016 | 576 | 0 | 0 | 0 | 0 | 0 | 0 | 0 | 0 | 0 | 0 | 0 | 0 |
| 9019 | 579 | 2 | 0 | 0 | 0 | 0 | 0 | 0 | 0 | 0 | 0 | 1 | 6 |
| 9021 | 581 | 0 | 0 | 0 | 0 | 0 | 0 | 228 | 0 | 9 | 2 | 0 | 0 |
| 9022 | 582 | 28 | 0 | 0 | 0 | 0 | 0 | 0 | 0 | 0 | 0 | 0 | 0 |
| 9023 | 583 | 31 | 0 | 0 | 0 | 0 | 0 | 0 | 0 | 0 | 2 | 0 | 0 |
| 9025 | 585 | 14 | 0 | 0 | 0 | 0 | 0 | 0 | 0 | 0 | 29 | 0 | 0 |
| 9029 | 589 | 0 | 0 | 0 | 0 | 0 | 0 | 0 | 0 | 0 | 0 | 334 | 0 |
| 9030 | 590 | 47 | 0 | 0 | 0 | 0 | 0 | 0 | 0 | 0 | 471 | 0 | 0 |
| 9031 | 591 | 0 | 0 | 0 | 0 | 0 | 0 | 0 | 0 | 0 | 0 | 0 | 0 |
| 9033 | 593 | 0 | 0 | 0 | 0 | 0 | 0 | 0 | 0 | 0 | 0 | 0 | 0 |
| 9034 | 594 | 0 | 0 | 0 | 0 | 0 | 0 | 0 | 0 | 0 | 0 | 0 | 0 |
| 9037 | 597 | 13 | 0 | 0 | 0 | 0 | 3 | 0 | 0 | 0 | 0 | 0 | 0 |
| 9040 | 600 | 1 | 0 | 0 | 0 | 0 | 0 | 0 | 0 | 0 | 0 | 0 | 0 |
| 9042 | 602 | 11 | 0 | 0 | 0 | 0 | 0 | 0 | 0 | 0 | 0 | 0 | 0 |
| 9044 | 604 | 1 | 0 | 0 | 0 | 0 | 0 | 0 | 0 | 0 | 0 | 0 | 0 |
| 9045 | 605 | 1 | 0 | 0 | 0 | 0 | 0 | 0 | 0 | 0 | 0 | 0 | 0 |
| 9047 | 800 | 5 | 1 | 0 | 0 | 0 | 0 | 0 | 0 | 0 | 0 | 0 | 0 |
| 9065 | 624 | 1 | 0 | 0 | 0 | 0 | 0 | 0 | 0 | 0 | 46 | 0 | 0 |
| 9070 | 629 | 1 | 0 | 0 | 0 | 0 | 0 | 0 | 0 | 0 | 0 | 0 | 0 |
| 9084 | 643 | 1 | 0 | 0 | 0 | 0 | 0 | 0 | 8 | 0 | 0 | 0 | 0 |
| 9086 | 645 | 0 | 0 | 0 | 0 | 0 | 0 | 0 | 0 | 0 | 0 | 0 | 0 |
| 9089 | 648 | 0 | 0 | 0 | 0 | 0 | 0 | 0 | 0 | 0 | 0 | 0 | 0 |
| 9100 | 658 | 26 | 0 | 0 | 0 | 0 | 0 | 0 | 0 | 0 | 0 | 0 | 0 |
| 9109 | 667 | 0 | 0 | 0 | 0 | 0 | 0 | 0 | 0 | 0 | 0 | 0 | 0 |
| 9112 | 670 | 0 | 0 | 0 | 0 | 0 | 0 | 0 | 0 | 0 | 0 | 0 | 0 |
| 9119 | 677 | 2 | 0 | 0 | 0 | 0 | 0 | 0 | 0 | 0 | 0 | 0 | 0 |
| 9126 | 684 | 3 | 0 | 0 | 0 | 0 | 0 | 0 | 0 | 0 | 2 | 0 | 0 |
| 9138 | 696 | 0 | 0 | 0 | 0 | 0 | 0 | 0 | 0 | 0 | 0 | 0 | 0 |
| 9139 | 697 | 0 | 0 | 0 | 0 | 0 | 0 | 0 | 0 | 0 | 0 | 0 | 0 |
| 9153 | 708 | 0 | 0 | 0 | 0 | 0 | 0 | 0 | 0 | 0 | 0 | 0 | 0 |
| 9154 | 709 | 5 | 0 | 0 | 0 | 0 | 0 | 0 | 0 | 0 | 0 | 0 | 0 |
| 9155 | 710 | 0 | 0 | 0 | 0 | 1 | 0 | 0 | 0 | 0 | 0 | 0 | 0 |
| 9156 | 711 | 0 | 0 | 0 | 0 | 0 | 0 | 0 | 0 | 0 | 0 | 0 | 0 |
| 9157 | 712 | 1 | 0 | 0 | 0 | 0 | 0 | 0 | 0 | 0 | 0 | 0 | 0 |
| 9160 | 714 | 2 | 0 | 0 | 0 | 0 | 0 | 0 | 0 | 0 | 0 | 0 | 0 |
| 9161 | 715 | 0 | 0 | 0 | 0 | 0 | 0 | 0 | 0 | 0 | 0 | 0 | 0 |
| 9170 | 724 | 6 | 0 | 0 | 0 | 0 | 0 | 0 | 0 | 0 | 0 | 0 | 0 |
| 9171 | 725 | 0 | 0 | 0 | 0 | 0 | 0 | 0 | 0 | 0 | 0 | 0 | 0 |
| 9172 | 726 | 0 | 0 | 0 | 0 | 0 | 0 | 0 | 34 | 0 | 0 | 0 | 0 |
| 9193 | 732 | 3 | 0 | 0 | 0 | 0 | 0 | 0 | 0 | 0 | 0 | 0 | 0 |
| 9204 | 736 | 3 | 0 | 0 | 0 | 0 | 0 | 0 | 0 | 0 | 0 | 0 | 0 |
| Subject ID | Tube ID | Parechovirus | Pestivirus | Pneumovirus | Polyomavirus | Respirovirus | Rhinovirus | Roseolovirus |  | | | | |
| 9006 | 566 | 0 | 0 | 0 | 0 | 0 | 0 | 0 |  | | | | |
| 9007 | 567 | 0 | 0 | 0 | 0 | 0 | 0 | 5 |  | | | | |
| 9008 | 568 | 3 | 0 | 0 | 0 | 0 | 0 | 0 |  | | | | |
| 9009 | 569 | 0 | 0 | 0 | 0 | 0 | 0 | 0 |  | | | | |
| 9011 | 571 | 0 | 0 | 0 | 0 | 7 | 21 | 0 |  | | | | |
| 9012 | 572 | 0 | 0 | 0 | 0 | 0 | 0 | 0 |  | | | | |
| 9013 | 573 | 0 | 0 | 0 | 0 | 347 | 435 | 0 |  | | | | |
| 9014 | 574 | 0 | 0 | 0 | 0 | 0 | 0 | 0 |  | | | | |
| 9015 | 575 | 0 | 0 | 0 | 0 | 0 | 0 | 0 |  | | | | |
| 9016 | 576 | 0 | 0 | 0 | 0 | 0 | 0 | 0 |  | | | | |
| 9019 | 579 | 0 | 0 | 470 | 0 | 0 | 0 | 0 |  | | | | |
| 9021 | 581 | 0 | 0 | 0 | 0 | 0 | 0 | 0 |  | | | | |
| 9022 | 582 | 0 | 0 | 0 | 0 | 0 | 0 | 0 |  | | | | |
| 9023 | 583 | 0 | 0 | 0 | 1 | 0 | 0 | 0 |  | | | | |
| 9025 | 585 | 0 | 0 | 0 | 3 | 0 | 0 | 0 |  | | | | |
| 9029 | 589 | 0 | 0 | 0 | 0 | 0 | 0 | 0 |  | | | | |
| 9030 | 590 | 0 | 0 | 0 | 0 | 337 | 0 | 0 |  | | | | |
| 9031 | 591 | 0 | 0 | 0 | 0 | 0 | 2 | 0 |  | | | | |
| 9033 | 593 | 0 | 0 | 0 | 0 | 0 | 9 | 0 |  | | | | |
| 9034 | 594 | 0 | 0 | 0 | 0 | 0 | 0 | 0 |  | | | | |
| 9037 | 597 | 0 | 0 | 0 | 0 | 0 | 0 | 0 |  | | | | |
| 9040 | 600 | 0 | 0 | 0 | 0 | 0 | 0 | 0 |  | | | | |
| 9042 | 602 | 0 | 0 | 0 | 0 | 0 | 0 | 0 |  | | | | |
| 9044 | 604 | 0 | 0 | 1 | 0 | 0 | 0 | 0 |  | | | | |
| 9045 | 605 | 0 | 0 | 0 | 0 | 0 | 5 | 0 |  | | | | |
| 9047 | 800 | 0 | 0 | 0 | 0 | 0 | 0 | 0 |  | | | | |
| 9065 | 624 | 0 | 0 | 0 | 0 | 0 | 0 | 0 |  | | | | |
| 9070 | 629 | 0 | 0 | 0 | 0 | 0 | 0 | 0 |  | | | | |
| 9084 | 643 | 0 | 0 | 0 | 0 | 0 | 233 | 0 |  | | | | |
| 9086 | 645 | 0 | 0 | 0 | 0 | 0 | 2 | 0 |  | | | | |
| 9089 | 648 | 0 | 0 | 0 | 0 | 0 | 4 | 0 |  | | | | |
| 9100 | 658 | 0 | 0 | 0 | 0 | 0 | 0 | 0 |  | | | | |
| 9109 | 667 | 0 | 0 | 0 | 0 | 0 | 0 | 0 |  | | | | |
| 9112 | 670 | 0 | 2 | 0 | 0 | 0 | 0 | 0 |  | | | | |
| 9119 | 677 | 0 | 0 | 0 | 0 | 0 | 0 | 0 |  | | | | |
| 9126 | 684 | 0 | 0 | 0 | 0 | 0 | 0 | 0 |  | | | | |
| 9138 | 696 | 0 | 0 | 0 | 0 | 0 | 0 | 0 |  | | | | |
| 9139 | 697 | 0 | 0 | 0 | 0 | 0 | 0 | 0 |  | | | | |
| 9153 | 708 | 0 | 0 | 0 | 0 | 0 | 0 | 0 |  | | | | |
| 9154 | 709 | 0 | 0 | 0 | 0 | 0 | 0 | 0 |  | | | | |
| 9155 | 710 | 60 | 0 | 0 | 0 | 0 | 0 | 0 |  | | | | |
| 9156 | 711 | 0 | 0 | 0 | 0 | 0 | 0 | 0 |  | | | | |
| 9157 | 712 | 0 | 0 | 0 | 0 | 0 | 0 | 0 |  | | | | |
| 9160 | 714 | 0 | 0 | 0 | 0 | 0 | 0 | 0 |  | | | | |
| 9161 | 715 | 0 | 0 | 0 | 0 | 0 | 0 | 0 |  | | | | |
| 9170 | 724 | 0 | 0 | 0 | 0 | 0 | 1 | 0 |  | | | | |
| 9171 | 725 | 5 | 0 | 0 | 0 | 0 | 0 | 0 |  | | | | |
| 9172 | 726 | 0 | 0 | 0 | 0 | 0 | 523 | 0 |  | | | | |
| 9193 | 732 | 0 | 0 | 0 | 0 | 0 | 7 | 0 |  | | | | |
| 9204 | 736 | 0 | 0 | 0 | 0 | 0 | 1 | 0 |  | | | | |

NP - Afebrile

| Subject ID | Tube ID | Alphapapillomavirus | Anellovirus | Bocavirus | Coronavirus | Enterovirus | Hepacivirus | Mastadenovirus | Metapneumovirus | Pneumovirus | Respirovirus | Rhinovirus | Roseolovirus |
| --- | --- | --- | --- | --- | --- | --- | --- | --- | --- | --- | --- | --- | --- |
| 9050 | 609 | 0 | 0 | 0 | 0 | 0 | 0 | 0 | 0 | 0 | 0 | 0 | 0 |
| 9051 | 610 | 0 | 3 | 0 | 0 | 0 | 0 | 0 | 0 | 0 | 0 | 0 | 0 |
| 9054 | 613 | 0 | 0 | 0 | 0 | 0 | 0 | 0 | 0 | 0 | 0 | 0 | 0 |
| 9055 | 614 | 0 | 0 | 0 | 0 | 1 | 0 | 0 | 0 | 0 | 0 | 6 | 0 |
| 9056 | 615 | 0 | 0 | 0 | 0 | 0 | 0 | 0 | 0 | 2 | 0 | 0 | 0 |
| 9057 | 616 | 0 | 0 | 0 | 0 | 0 | 0 | 0 | 0 | 0 | 0 | 0 | 0 |
| 9059 | 618 | 0 | 0 | 0 | 0 | 0 | 0 | 0 | 0 | 0 | 0 | 0 | 0 |
| 9060 | 619 | 0 | 6 | 0 | 0 | 0 | 0 | 0 | 0 | 0 | 0 | 0 | 0 |
| 9061 | 620 | 0 | 0 | 0 | 0 | 0 | 0 | 0 | 0 | 0 | 0 | 0 | 0 |
| 9062 | 621 | 0 | 0 | 0 | 0 | 0 | 0 | 0 | 0 | 0 | 0 | 0 | 0 |
| 9063 | 622 | 0 | 3 | 0 | 0 | 2 | 0 | 0 | 0 | 0 | 0 | 10 | 0 |
| 9066 | 625 | 0 | 0 | 0 | 2 | 0 | 0 | 0 | 0 | 0 | 0 | 0 | 0 |
| 9067 | 626 | 0 | 1 | 0 | 0 | 0 | 0 | 0 | 0 | 0 | 3 | 0 | 0 |
| 9071 | 630 | 0 | 0 | 0 | 0 | 0 | 0 | 0 | 0 | 0 | 0 | 0 | 0 |
| 9072 | 631 | 0 | 0 | 0 | 0 | 0 | 0 | 0 | 0 | 0 | 0 | 0 | 0 |
| 9075 | 634 | 0 | 0 | 0 | 0 | 0 | 0 | 0 | 0 | 0 | 0 | 1 | 0 |
| 9076 | 635 | 0 | 0 | 0 | 0 | 0 | 0 | 0 | 0 | 0 | 0 | 0 | 0 |
| 9080 | 639 | 0 | 0 | 0 | 0 | 0 | 0 | 0 | 0 | 0 | 0 | 1 | 0 |
| 9081 | 640 | 0 | 6 | 0 | 0 | 0 | 0 | 0 | 0 | 0 | 0 | 0 | 0 |
| 9082 | 641 | 0 | 0 | 0 | 0 | 0 | 0 | 0 | 0 | 0 | 0 | 0 | 0 |
| 9083 | 642 | 0 | 0 | 0 | 0 | 0 | 0 | 0 | 0 | 0 | 0 | 0 | 0 |
| 9085 | 644 | 0 | 2 | 0 | 0 | 1 | 0 | 0 | 0 | 0 | 0 | 35 | 0 |
| 9087 | 646 | 0 | 0 | 0 | 0 | 35 | 0 | 0 | 0 | 0 | 0 | 6115 | 0 |
| 9088 | 647 | 0 | 73 | 0 | 0 | 0 | 0 | 0 | 0 | 0 | 0 | 0 | 0 |
| 9090 | 649 | 0 | 0 | 0 | 0 | 0 | 0 | 0 | 0 | 0 | 0 | 6 | 0 |
| 9091 | 650 | 0 | 0 | 0 | 0 | 0 | 1 | 0 | 0 | 0 | 0 | 0 | 0 |
| 9093 | 652 | 0 | 8 | 0 | 0 | 0 | 0 | 0 | 0 | 0 | 0 | 2 | 0 |
| 9094 | 653 | 0 | 0 | 0 | 0 | 0 | 0 | 0 | 0 | 0 | 0 | 8 | 0 |
| 9095 | 799 | 0 | 0 | 0 | 0 | 0 | 0 | 2 | 0 | 0 | 0 | 0 | 0 |
| 9096 | 654 | 0 | 0 | 0 | 0 | 0 | 0 | 0 | 0 | 0 | 0 | 0 | 0 |
| 9097 | 655 | 0 | 0 | 0 | 0 | 0 | 0 | 0 | 0 | 0 | 0 | 0 | 0 |
| 9098 | 656 | 0 | 28 | 0 | 0 | 0 | 0 | 0 | 0 | 0 | 0 | 0 | 0 |
| 9099 | 657 | 0 | 2 | 0 | 0 | 0 | 0 | 0 | 0 | 0 | 0 | 0 | 0 |
| 9101 | 659 | 0 | 0 | 0 | 0 | 0 | 0 | 0 | 0 | 0 | 0 | 0 | 0 |
| 9102 | 660 | 0 | 0 | 0 | 0 | 0 | 0 | 0 | 0 | 0 | 0 | 0 | 0 |
| 9103 | 661 | 0 | 0 | 0 | 0 | 0 | 0 | 0 | 0 | 0 | 0 | 0 | 0 |
| 9106 | 664 | 0 | 9 | 2 | 0 | 0 | 0 | 0 | 0 | 0 | 0 | 0 | 0 |
| 9107 | 665 | 0 | 4 | 0 | 0 | 0 | 0 | 0 | 0 | 0 | 0 | 0 | 0 |
| 9110 | 668 | 0 | 0 | 0 | 0 | 0 | 0 | 0 | 0 | 0 | 0 | 0 | 0 |
| 9111 | 669 | 0 | 1 | 0 | 0 | 0 | 0 | 0 | 0 | 0 | 0 | 0 | 0 |
| 9113 | 671 | 0 | 0 | 0 | 0 | 0 | 0 | 0 | 0 | 0 | 0 | 39 | 0 |
| 9114 | 672 | 0 | 0 | 0 | 0 | 0 | 0 | 8 | 0 | 0 | 0 | 0 | 0 |
| 9115 | 673 | 0 | 0 | 0 | 0 | 0 | 0 | 0 | 0 | 0 | 0 | 0 | 0 |
| 9116 | 674 | 0 | 0 | 0 | 0 | 0 | 0 | 2 | 0 | 0 | 0 | 0 | 0 |
| 9117 | 675 | 0 | 0 | 0 | 0 | 0 | 0 | 0 | 0 | 0 | 0 | 0 | 0 |
| 9118 | 676 | 0 | 0 | 0 | 0 | 502 | 0 | 0 | 0 | 0 | 0 | 198 | 0 |
| 9121 | 679 | 0 | 0 | 0 | 0 | 0 | 0 | 0 | 0 | 0 | 0 | 0 | 0 |
| 9122 | 680 | 0 | 0 | 0 | 0 | 0 | 0 | 0 | 0 | 0 | 0 | 0 | 0 |
| 9123 | 681 | 0 | 0 | 0 | 0 | 3 | 0 | 0 | 0 | 0 | 0 | 0 | 0 |
| 9125 | 683 | 0 | 0 | 0 | 0 | 0 | 0 | 0 | 0 | 0 | 0 | 0 | 0 |
| 9127 | 685 | 0 | 0 | 0 | 0 | 0 | 0 | 0 | 0 | 0 | 0 | 0 | 0 |
| 9128 | 686 | 0 | 67 | 0 | 0 | 0 | 0 | 0 | 0 | 0 | 0 | 6 | 0 |
| 9129 | 687 | 0 | 0 | 0 | 0 | 0 | 0 | 0 | 0 | 0 | 0 | 0 | 0 |
| 9130 | 688 | 0 | 1 | 0 | 0 | 0 | 0 | 0 | 0 | 0 | 0 | 0 | 0 |
| 9131 | 689 | 0 | 0 | 0 | 0 | 0 | 0 | 0 | 0 | 0 | 0 | 0 | 0 |
| 9132 | 690 | 0 | 0 | 0 | 0 | 0 | 0 | 0 | 0 | 0 | 0 | 0 | 0 |
| 9133 | 691 | 0 | 0 | 0 | 0 | 0 | 0 | 0 | 0 | 0 | 0 | 0 | 0 |
| 9134 | 692 | 0 | 0 | 0 | 0 | 0 | 0 | 0 | 0 | 0 | 0 | 0 | 0 |
| 9135 | 693 | 0 | 0 | 0 | 0 | 0 | 0 | 0 | 0 | 0 | 0 | 0 | 0 |
| 9136 | 694 | 0 | 3 | 0 | 0 | 0 | 0 | 0 | 0 | 0 | 0 | 0 | 0 |
| 9137 | 695 | 0 | 0 | 0 | 0 | 0 | 0 | 0 | 0 | 0 | 0 | 0 | 0 |
| 9142 | 699 | 0 | 0 | 0 | 0 | 0 | 0 | 0 | 0 | 0 | 0 | 0 | 0 |
| 9144 | 701 | 0 | 15 | 0 | 0 | 0 | 0 | 0 | 0 | 0 | 0 | 0 | 2 |
| 9145 | 702 | 0 | 1 | 0 | 0 | 0 | 0 | 0 | 0 | 0 | 0 | 16 | 0 |
| 9146 | 703 | 0 | 0 | 0 | 0 | 0 | 0 | 0 | 0 | 0 | 0 | 0 | 0 |
| 9147 | 704 | 0 | 1 | 0 | 0 | 0 | 0 | 0 | 0 | 0 | 0 | 0 | 0 |
| 9149 | 706 | 0 | 6 | 0 | 0 | 0 | 0 | 0 | 0 | 0 | 0 | 0 | 0 |
| 9150 | 480 | 0 | 0 | 0 | 0 | 0 | 0 | 0 | 0 | 0 | 0 | 2 | 0 |
| 9151 | 484 | 0 | 0 | 0 | 0 | 4 | 0 | 0 | 0 | 0 | 0 | 83 | 0 |
| 9152 | 707 | 0 | 0 | 0 | 0 | 0 | 0 | 0 | 0 | 0 | 0 | 0 | 0 |
| 9159 | 713 | 0 | 0 | 0 | 0 | 0 | 0 | 0 | 0 | 0 | 0 | 0 | 0 |
| 9162 | 716 | 1 | 0 | 0 | 0 | 0 | 0 | 0 | 0 | 0 | 0 | 0 | 0 |
| 9163 | 717 | 0 | 2 | 0 | 0 | 2 | 0 | 0 | 0 | 0 | 0 | 2 | 0 |
| 9166 | 720 | 0 | 0 | 0 | 0 | 0 | 0 | 0 | 0 | 0 | 0 | 0 | 0 |
| 9168 | 722 | 0 | 3 | 0 | 0 | 0 | 0 | 0 | 0 | 0 | 0 | 0 | 0 |
| 9179 | 727 | 0 | 0 | 0 | 0 | 0 | 0 | 0 | 0 | 0 | 0 | 0 | 54 |
| 9182 | 729 | 0 | 0 | 0 | 0 | 0 | 0 | 0 | 0 | 0 | 0 | 0 | 0 |
| 9184 | 730 | 0 | 4 | 0 | 0 | 0 | 0 | 0 | 0 | 0 | 0 | 0 | 0 |
| 9187 | 731 | 0 | 8 | 0 | 0 | 0 | 0 | 0 | 0 | 0 | 0 | 0 | 0 |
| 9194 | 733 | 0 | 0 | 0 | 0 | 0 | 0 | 0 | 0 | 0 | 0 | 0 | 0 |
| 9195 | 734 | 0 | 1 | 0 | 0 | 0 | 0 | 0 | 0 | 0 | 0 | 0 | 2 |
